# Digital Design of Kurtosis-Controlled Ti-6Al-4V Lattices for Patient-Specific Orthopedic Implants: A Computational Framework

**DOI:** 10.3390/bioengineering13080934

**Published:** 2026-08-18

**Authors:** Marzhan Sadenova, Boris Syrnev, Bagdat Azamatov

**Affiliations:** Center of Excellence «VERITAS», D. Serikbayev East Kazakhstan Technical University, Ust-Kamenogorsk 070000, Kazakhstan; bsyrnev@edu.ektu.kz (B.S.); bazamatov@edu.ektu.kz (B.A.)

**Keywords:** Ti-6Al-4V, porous lattice, structural kurtosis, orthopedic implants, patient-specific implants, additive manufacturing, selective laser melting, CAD reconstruction, finite-element analysis, GAN-CAD-FEA, Gibson–Ashby scaling, computational design framework, lattice benchmarking

## Abstract

Porous Ti-6Al-4V lattice implants combine high specific strength, osseointegrative porosity, and compatibility with additive manufacturing, but conventional stiffness tuning through porosity, pore size, or unit-cell topology compromises biological pore requirements. This study presents a computational design framework in which structural kurtosis, the normalized interlayer offset between neighboring layers of a periodic cubic lattice, regulates elastic response at fixed global porosity. Closed-form expressions for the effective modulus are derived from first principles: the aligned configuration from the axial load-bearing area fraction, and the interlayer-shifted configuration from Euler–Bernoulli beam theory for guided-end connecting members. The derivations reproduce the Gibson–Ashby exponents *n* = 1 and *n* = 2, replacing the previously asserted power law, and a calibrated one-parameter interpolation bridges intermediate offsets. At 65% porosity, the effective modulus falls from 16.5 GPa in the aligned lattice to 2.64 GPa in the shifted lattice. A local-yield analysis based on peak bending curvature gives recoverable elastic strains of 1.37% at 89% porosity and 0.68% at 65%; the compliance-based values of 20.5% and 5.12% are kinematic upper bounds that neglect plastic hinging. A prefactor-free benchmark shows that obtaining the same 6.25-fold reduction by increased porosity alone would require 85.9–94.4% porosity and 0.17–0.28 mm struts, outside the osseointegration window and the resolution of selective laser melting. A GAN-CAD-FEA workflow reproduced the analytical moduli to within 7% across six design cases. All results are analytical and numerical; no specimens were fabricated or tested, and experimental validation remains required.

## 1. Introduction

Patient-specific orthopedic reconstruction increasingly requires implants that combine biological integration with mechanical compatibility in the local bone environment. Porous metallic lattices have become one of the most promising design strategies for this purpose because they allow dense load-bearing alloys to be transformed into structures with reduced stiffness, interconnected porosity, and controlled mass transport pathways. Among clinically relevant metallic biomaterials, Ti-6Al-4V remains a dominant alloy for orthopedic and spinal applications due to its high strength-to-weight ratio, corrosion resistance, fatigue performance, and compatibility with laser powder-bed fusion and selective laser melting technologies [1,2,3,4,5]. Additive manufacturing has expanded implant design from conventional porous coatings to fully three-dimensional lattice architectures with programmable porosity, pore size, topology, and graded mechanical properties [5,6,7,8].

Despite this progress, the central challenge in orthopedic implant design remains the simultaneous satisfaction of biological and mechanical requirements. Bone ingrowth and long-term fixation require an interconnected pore network with sufficient pore size, permeability, and surface area for vascularization, osteoblast migration, and osseointegration [6,7,8,9,10]. At the same time, the implant must transfer load in a manner compatible with the surrounding cortical or trabecular bone. Dense Ti-6Al-4V has an elastic modulus above 100 GPa, whereas trabecular bone may exhibit moduli below a few gigapascals, and cortical bone occupies a higher but still substantially lower range [11,12,13]. This modulus mismatch promotes stress shielding, load-transfer imbalance, interfacial micromotion, bone resorption, and eventual loss of fixation [14,15]. Porous titanium lattices reduce this mismatch, but reducing stiffness by increasing porosity may also weaken the structure and alter the pore architecture required for biological integration.

Current approaches to mechanical tuning of porous implants generally follow three routes: changing porosity, changing unit-cell topology, or introducing compliant and spring-like structural features. Porosity modification is straightforward and effective, but it couples stiffness reduction to changes in pore volume, strut thickness, surface area, and load-bearing cross-section. Topology modification, including cubic, diamond, gyroid, and other periodic or triply periodic minimal surface structures, can broaden the accessible range of stiffness and permeability, but topology transitions may introduce discontinuities, stress concentrations, and manufacturing complexity [16,17,18,19,20]. Compliant or spring-like designs can improve deformation capacity; however, they are often difficult to integrate into spatially graded patient-specific implants where stiffness must change continuously along a bone–implant interface. A remaining design gap is the lack of a simple geometric parameter that can tune elastic response locally while preserving the global porosity required for osseointegration.

Recent work has continued to expand this design space, and it is against that recent body of evidence that the present contribution should be positioned. Studies published in the last three years have characterized the compressive, tensile, and fatigue response of laser powder-bed fusion Ti-6Al-4V lattices and quantified the influence of build orientation, defect population, and surface texture on their as-built properties [21,22,23], and have also mapped the relationship between pore-size parameters and osseointegration outcomes in orthopedic applications [24]. Comparative studies of body-centered-cubic, triply periodic minimal surface, and auxetic Ti-6Al-4V architectures have further widened the reported range of achievable moduli and deformation modes, although in every case the achievable stiffness remains coupled to the relative density of the structure [25,26,27,28]. In parallel, post-processing routes such as electropolishing have been shown to modify the effective load-bearing cross-section of thin struts [29], and machine learning approaches, including generative adversarial networks conditioned on process parameters, have been applied to the inverse design of lattice and triply periodic minimal surface architectures [30,31]. Together, these contributions confirm both the interest in geometrically programmable metallic lattices and the persistent gap between idealized design models and as-built mechanical behavior, which motivates the explicitly computational framing adopted in the present work.

This study addresses this research gap by introducing structural kurtosis as a lattice-level design parameter for porous Ti-6Al-4V implants. In the present work, structural kurtosis is not used in the statistical sense. The term is used here to describe the controlled skewing of the lattice-layer arrangement rather than a distributional property. It is defined as the normalized interlayer offset between neighboring layers of a periodic cubic lattice, K=Δ/a, where Δ is the relative layer shift and a is the unit-cell size. This parameter describes the controlled geometric skewing of the lattice. By shifting adjacent layers while maintaining the same nominal pore volume, the load path changes from predominantly axial stretching to a combined axial-bending deformation mode. This transition provides a direct route to reduce the effective modulus and increase elastic strain capacity without redesigning the unit-cell topology or sacrificing the global pore-volume conditions required for tissue ingrowth.

The proposed concept is particularly relevant for patient-specific orthopedic implants, where bone quality varies with anatomical site, age, pathology, and local loading conditions. A single implant may contact cortical shell regions, trabecular zones, and transitional interfaces that require different stiffness levels. A design parameter such as structural kurtosis can therefore provide local mechanical programming within a common lattice family. This is important for personalized orthopedic repair because it allows stiffness adaptation to be achieved through geometric control rather than through abrupt topology switching or biologically unfavorable porosity changes.

In addition to the analytical formulation, this study integrates structural kurtosis into a digital design workflow based on conditional generative adversarial networks, CAD reconstruction, and finite-element validation. GAN-based topology generation provides candidate lattice configurations for target porosity and stiffness conditions, CAD reconstruction converts voxel-based geometries into manufacturable implant models, and finite-element simulation verifies the predicted elastic response. This GAN-CAD-FEA workflow connects the analytical design rule with virtual mechanical testing, enabling rapid generation and evaluation of patient-specific lattice implants [32,33,34,35,36,37,38].

The objectives of this study are therefore as follows: (i) to derive, from axial equilibrium and beam-bending mechanics rather than by curve fitting, an analytical framework linking porosity and structural kurtosis to the elastic behavior of cubic Ti-6Al-4V lattices; (ii) to obtain quantitative relationships for the effective modulus and for the recoverable elastic strain that remains admissible once local bending stress and plastic hinging are accounted for; (iii) to identify the mechanical tuning range available at porosity levels relevant to osseointegration; (iv) to benchmark the resulting design space quantitatively against established lattice topologies in terms of stiffness range, strain capacity, and manufacturability; (v) to quantify the sensitivity of the predicted moduli to as-built strut-thickness deviations typical of selective laser melting; and (vi) to demonstrate the use of a GAN-CAD-FEA digital pipeline for transforming the analytical model into patient-specific lattice candidates. It should be stated at the outset that the present study is entirely computational. All mechanical values reported are analytical or finite-element predictions, and no specimens were fabricated, imaged, or tested. The work therefore establishes a computational design framework rather than a demonstrated design methodology, and every claim in this manuscript should be read in that light.

## 2. Materials and Methods

### 2.1. Lattice Geometry and Porosity Definition

The computational framework was built around a periodic cubic Ti-6Al-4V lattice composed of bar-like structural members arranged along the principal axes of a repeating unit cell. This geometry was selected deliberately: it is simple enough for analytical treatment, regular enough for parametric reconstruction, and compatible with selective laser melting and laser powder-bed fusion manufacturing. The unit-cell size is denoted by a, while the lattice-thickness coefficient is represented by the nondimensional parameter m. In this model, increasing m increases the effective strut thickness and reduces the void fraction of the lattice.

Porosity P was defined as the void-volume fraction of the unit cell and was calculated from the solid-volume complement of the repeated bar network. Five porosity levels were evaluated: 89%, 65%, 35%, 10.4%, and 0%. The 0% porosity condition was included only as the dense Ti-6Al-4V reference limit. These conditions cover the transition from highly porous scaffold-like lattices to the limiting dense Ti-6Al-4V case. The corresponding strut thicknesses for practical unit-cell sizes are reported in Table 1, which defines the geometric input space used in the subsequent mechanical analysis.

As shown in Table 1, the same nondimensional thickness coefficient produces different absolute strut dimensions depending on the selected unit-cell size. This relationship is important for additive manufacturing because the minimum printable strut size constrains the smallest feasible cell geometry. The 65% porosity case was treated as a key design condition because it provides a useful compromise between mechanical continuity and open pore space for bone ingrowth and vascularization.

**Figure 1:**
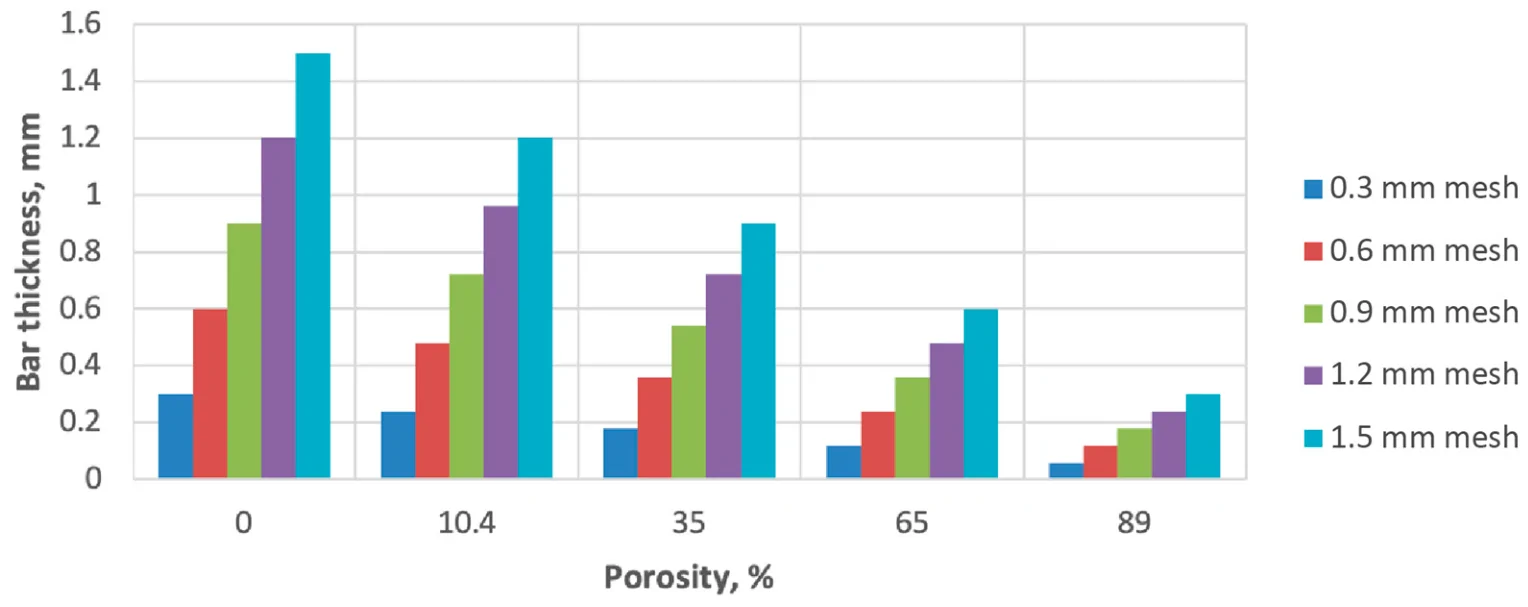
Strut thickness as a function of lattice porosity for different unit-cell sizes.

The same geometric relationship is visualized in Figure 1. The figure makes the manufacturing implication clear: larger unit cells require proportionally thicker struts to maintain the same porosity state, whereas smaller cells allow finer structural features but may approach process-resolution limits.

Together, Table 1 and Figure 1 establish the baseline design space for the study. All comparisons of effective modulus, recoverable deformation, Poisson response, and GAN-CAD-FEA validation were derived from this consistent relationship between unit-cell size, strut thickness, and porosity.

### 2.2. Structural Kurtosis and Two Deformation Modes

To isolate the mechanical role of interlayer offset, two idealized lattice configurations were compared. The first configuration, referred to as Type I, represents an aligned axial lattice in which neighboring layers remain vertically aligned and the main compressive load is transferred through direct stretching and compression of load-bearing members. This configuration is relatively stiff because the force path is short, straight, and predominantly axial.

**Figure 2:**
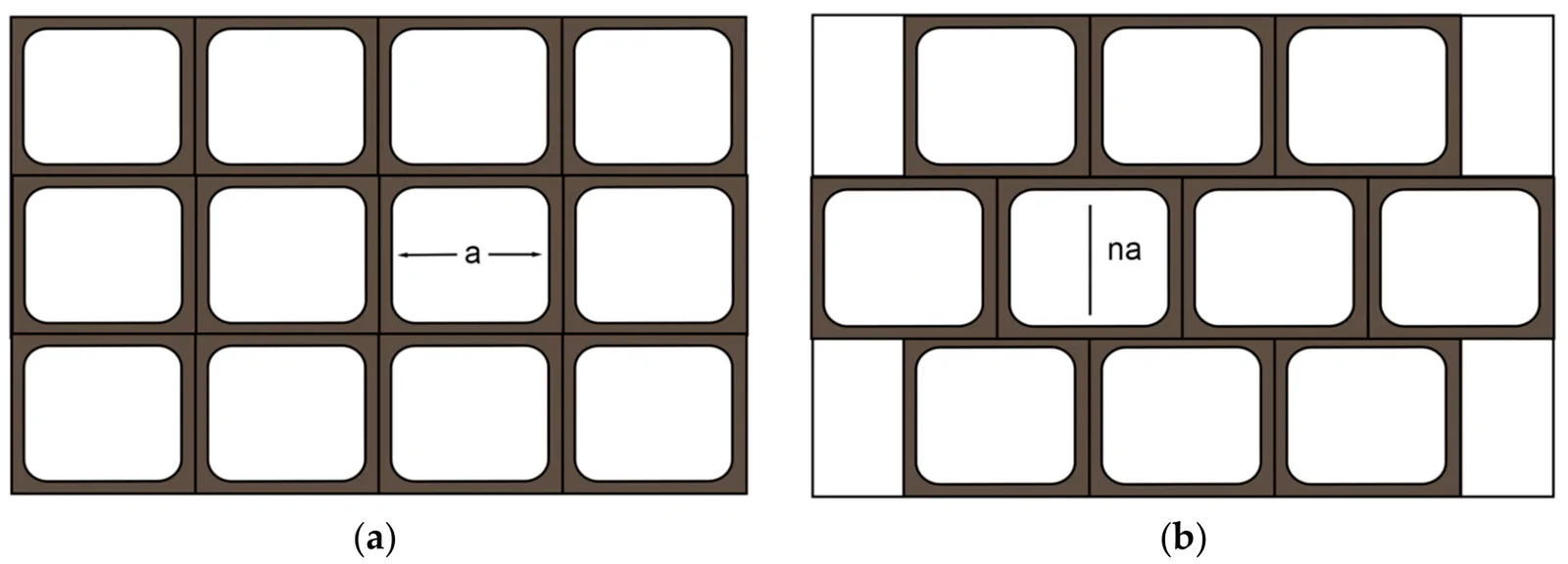
Schematic representation of the two lattice configurations: (**a**) aligned axial lattice and (**b**) interlayer-shifted kurtosis-controlled lattice. Here, (a) denotes the unit-cell size, and (na) denotes the lateral offset between neighboring lattice layers, where (n) is the normalized offset coefficient. The structural kurtosis used in the analytical model is defined as (K = Δ/a), where (Δ) is the corresponding lateral interlayer displacement.

The second configuration, referred to as Type II, represents an interlayer-shifted lattice in which adjacent horizontal layers are laterally displaced relative to each other. This displacement changes the load-transfer path and forces the connecting members to deform through a mixed axial-bending mechanism. The difference between these two deformation modes is shown schematically in Figure 2.

As shown in Figure 2, the aligned lattice preserves a direct vertical load path, whereas the shifted lattice introduces an offset that increases structural compliance. This change is the basis of the proposed stiffness-control strategy. Instead of reducing stiffness by increasing pore volume, the lattice can be made more compliant by changing the relative position of neighboring layers.

Structural kurtosis was defined as a dimensionless geometric descriptor in Equation (1):

K = Δ/a,(1)

where Δ is the lateral offset between neighboring lattice layers and a is the unit-cell size. Thus, K = 0 corresponds to fully aligned layers, whereas K = 0.5 corresponds to a half-cell offset. In this study, the term structural kurtosis does not refer to the statistical fourth moment. Instead, it is used as a geometric design parameter that controls the degree of interlayer offset and, consequently, the dominant deformation mode of the lattice.

The main purpose of introducing K was to separate mechanical tuning from porosity control. Conventional lattice design often reduces stiffness by increasing pore volume, but this can compromise the amount of solid material available for load transfer. In contrast, the kurtosis-controlled approach allows stiffness and recoverable deformation to be adjusted while maintaining the same global porosity. This distinction is important for bone-implant applications, where pore volume must remain sufficient for tissue ingrowth while the mechanical response must be matched to the surrounding bone.

### 2.3. Analytical Model: Mechanistic Derivation of the Effective Modulus

The analytical model was developed to provide a direct and physically interpretable relationship between lattice geometry and apparent mechanical response. Dense Ti-6Al-4V was used as the reference material, with Young’s modulus E_s_ = 103 GPa and a yield strength σ_y_ = 845 MPa. The model compares two limiting deformation regimes: an aligned axial regime, in which load transfer is dominated by stretching and compression of the vertical members, and an interlayer-shifted compliant regime, in which the connecting members transfer the load in bending. Both governing expressions are derived below from equilibrium and beam kinematics. Neither is obtained by fitting to numerical or experimental data, and neither contains an adjustable coefficient.

The unit cell of edge a contains three mutually orthogonal square-section bars of side t = 2 ma. Summing the three bar volumes and subtracting their pairwise intersections gives the relative density of the lattice and the corresponding porosity as Equation (2):

(2) $$\rho =3{\left(2m\right)}^{2}-2{\left(2m\right)}^{3}=1-P.$$

Equation (2) reproduces the porosity values of Table 1 exactly: m = 0.1, 0.2, 0.3, 0.4 and 0.5 give relative densities of 0.104, 0.352, 0.648, 0.896, and 1.000, that is, porosities of 89.6%, 64.8%, 35.2%, 10.4%, and 0%. The relative density is therefore not an independent input but a determined function of the single thickness coefficient m. This is what allows stiffness and porosity to be expressed on a common basis in the derivations that follow, and what makes the comparison with the Gibson–Ashby scaling laws direct.

**Figure 3:**
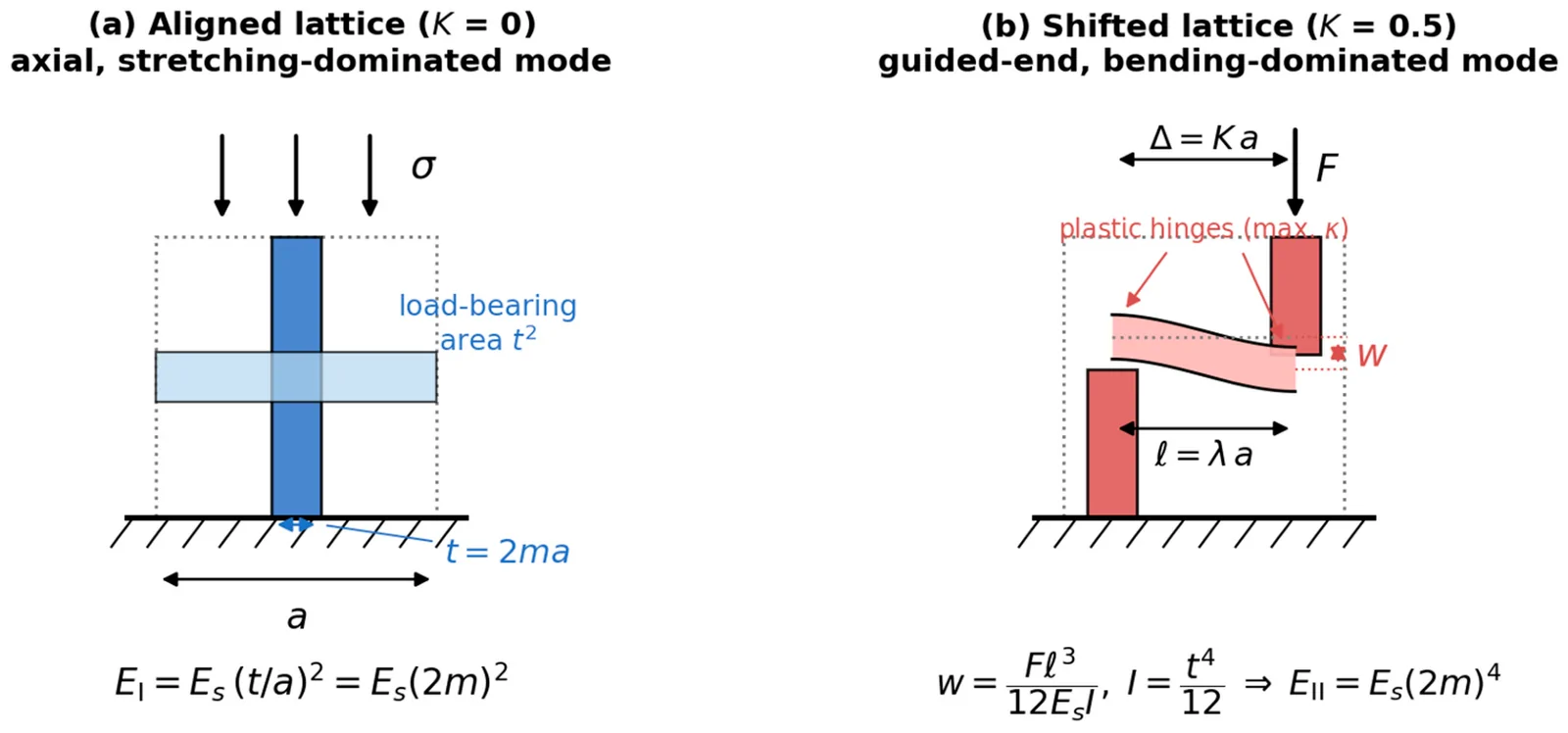
Mechanical idealization used to derive the governing equations: (**a**) the aligned lattice (K = 0), in which the applied stress is carried by the axial load-bearing area t^2^ of the vertical member, giving Equation (3); and (**b**) the interlayer-shifted lattice (K = 0.5), in which the offset forces the connecting member to act as a guided-end beam of span ℓ = λa, giving Equation (5). The positions of maximum curvature at the built-in ends, where local yielding and plastic hinging initiate, are indicated.

In the aligned configuration (K = 0, Figure 3a), the vertical bar of each cell is collinear with the vertical bar of the cell below it, so the compressive load is carried entirely by that bar. The horizontal bars are unloaded in the axial direction and act only as lateral ties. Under a uniform axial strain ε applied over the cell height a, the axial force in the loaded bar is F = E_s_t^2^ε, while the nominal stress referred to the full cell cross-section is σ = F/a^2^. Dividing the two gives the effective modulus of the Type I lattice as Equation (3):

(3) $$E_{I}=E_{s}{\left(t/a\right)}^{2}=E_{s}{\left(2m\right)}^{2}.$$

Equation (3) is a direct consequence of the load-bearing area fraction and contains no fitted parameter. Because the relative density of Equation (2) reduces to 3(2m)^2^ for slender struts, Equation (3) is equivalent to E_I_/E_s_ ≈ ρ/3; that is, a linear scaling with relative density with exponent *n* = 1. This is the classical Gibson–Ashby signature of a stretching-dominated architecture [13].

In the interlayer-shifted configuration (K = 0.5, Figure 3b), the vertical bars of successive layers are no longer collinear, so the axial load cannot pass straight down the structure. It must first be transferred laterally through the horizontal connecting bar. Because both ends of that bar are built into vertical members, it does not deform as a simply supported beam but as a guided-end beam: rotation is suppressed at both ends while a relative transverse displacement w develops between them. For a beam of span ℓ = λa and square section t × t, the guided-end deflection and the second moment of area are given by Equation (4):

(4) $$w=\frac{Fl^{3}}{12E_{s}I},\;I=\frac{t^{4}}{12},$$

where λ is the ratio of the effective bending span to the cell size. The applied stress referred to the cell cross-section is again σ = F/a^2^, and the corresponding global strain is ε = w/a, since the relative displacement w accumulates once per cell height. Substituting Equation (4) into these two relations and taking the base case λ = 1, in which the bending span equals the cell size, gives the effective modulus of the Type II lattice, as in Equation (5):

(5) $$E_{II}=E_{s}{\left(t/a\right)}^{4}=E_{s}{\left(2m\right)}^{4}.$$

The fourth-power exponent in Equation (5) therefore originates from the second moment of area of the bending member, I ∝ t^4^, and not from an empirical fit. The numerical prefactor is unity only because the guided-end boundary condition contributes a factor of 12 that cancels the factor 12 in I = t^4^/12; any other end condition changes that prefactor but leaves the exponent unchanged. Since the relative density scales as (2m)^2^ in the slender regime, Equation (5) is equivalent to E_II_/E_s_ ∝ ρ^2^, the classical Gibson–Ashby bending-dominated scaling with exponent *n* = 2 [13,17]. Equations (3) and (5) thus define the two mechanically bounding regimes of the kurtosis-controlled lattice family, K = 0 and K = 0.5, entirely in terms of standard structural mechanics. Intermediate values of K correspond to transitional states in which both mechanisms act simultaneously; these are treated by the interpolation introduced below and are evaluated numerically through the GAN-CAD-FEA workflow. The two idealizations from which Equations (3) and (5) follow are shown in Figure 3.

Equations (3) and (5) describe the two bounding configurations, but a patient-specific implant will generally require intermediate offsets, and the submitted version of this manuscript provided no model for them. The transition between a stretching-dominated and a bending-dominated load path is not amenable to a closed-form treatment for arbitrary K, because the two mechanisms act in parallel over a contact region whose extent depends on both K and the strut slenderness. The two bounds were therefore bridged by a one-parameter interpolation Equation (6):

(6) $$E\left(K\right)=E_{II}+\left(E_{I}-E_{II}\right){\left(1-2K\right)}^{q}.$$

where q is a transition exponent. Equation (6) is an interpolation and not a derivation, and it is labeled as such throughout: it is constructed to return Equation (3) at K = 0 and Equation (5) at K = 0.5, while q controls how rapidly the load path switches between the two regimes. The value q = 0.9 was obtained by calibration against the finite-element result for the single intermediate configuration examined here, 89% porosity at K = 0.3 (scenario A2 of Section 3.4), and it is the only calibrated quantity in the model; with q = 0.9, Equation (6) returns 1.90 GPa for that configuration. One calibration point is a weak constraint, and the sensitivity of intermediate-K predictions to q therefore remains an open item requiring a denser set of simulations and, ultimately, experiments. The design argument of this study rests on the two bounding values of Equations (3) and (5), which are derived and do not depend on q.

The recoverable elastic strain capacity was estimated using the elastic strain limit of dense Ti-6Al-4V as the reference. In the aligned axial lattice, the members carry only uniform axial stress, so the apparent elastic strain capacity coincides with the material-level elastic strain limit, as in Equation (7):

(7) $$\varepsilon_{I}=\frac{\sigma_{y}}{E_{s}}\approx 0.0082\;\left(0.82\%\right).$$

where ε_I_ is the elastic strain capacity of the aligned lattice and σ_y_ is the yield strength used for the Ti-6Al-4V model. For the interlayer-shifted lattice, global deformation is accommodated structurally through bending and rotation of the connecting members. If the local stress state within those members is disregarded and only the ratio of structural compliance to material compliance is considered, the apparent strain capacity appears to scale as Equation (8):

(8) $${\varepsilon_{II}}^{kin}=\frac{\varepsilon_{I}}{{\left(2m\right)}^{2}}.$$

Equation (8) is a purely kinematic upper bound. It expresses how much global strain the structure could accommodate if the constituent material were everywhere simultaneously at its axial elastic limit, and it is the expression from which the value of 20.5% at 89% porosity follows. It is not, however, an admissible elastic limit for a metallic lattice. In a bending-dominated member, the stress is not uniform across the section: it peaks at the outer fiber where the curvature is greatest, so yielding, and, subsequently, plastic hinging, begins there long before the global strain given by Equation (8) is reached.

To obtain a physically admissible limit, the peak local strain was evaluated from the beam curvature. For the guided-end beam of Equation (4), the bending moment is largest at the two built-in ends, where the curvature reaches κ_max_ = 6w/ℓ^2^ and the outer-fiber strain is ε_local_ = κ_max_t/2 = 3tw/ℓ^2^. Substituting ε = w/a and ℓ = λa gives ε_local_ = 3(2m)ε/λ^2^. Setting ε_local_ equal to the material elastic strain limit ε_I_ yields the local-yield-limited strain capacity of the shifted lattice, as shown in Equation (9):

(9) $${\varepsilon_{II}}^{loc}=\frac{\lambda^{2}\varepsilon_{I}}{3\left(2m\right)}.$$

The governing elastic strain capacity of the shifted lattice is then the smaller of the two limits, since the structure ceases to respond elastically as soon as either is exceeded, as in Equation (10):

(10) $$\varepsilon_{II}=min\left({\varepsilon_{II}}^{kin},\;{\varepsilon_{II}}^{loc}\right).$$

Equations (9) and (10) introduce precisely the local stress state that Equation (8) omits, and two consequences follow. First, the strain benefit of structural kurtosis exists only when 3(2m) < λ^2^; that is, only for sufficiently slender struts. In the base case λ = 1, the crossover occurs at 2m = 1/3, corresponding to a relative density of 0.259 or a porosity of 74.1%. Above that porosity the shifted lattice does tolerate more recoverable strain than the aligned lattice; below it, the local bending penalty outweighs the compliance gain. Second, the lattice yield stress implied by the model, σ* = E_II_ε_II_, is 2.3 MPa at 89% porosity and 18.0 MPa at 65% porosity. These values are of the same order as the compressive strengths reported for highly porous laser powder-bed fusion Ti-6Al-4V lattices [21,22], whereas Equation (8) would imply a recoverable regime far beyond any strength reported for this alloy system. The span ratio λ was retained explicitly because the effective bending span depends on how many cells a connecting member bridges; λ = 2 raises the local-yield limit by a factor of four, to 5.5% at 89% porosity, which brackets the uncertainty associated with this idealization.

Together, Equations (2)–(10) provide a compact analytical mapping between porosity, structural kurtosis, stiffness, and recoverable deformation, in which every exponent follows either from the load-bearing area fraction or from the second moment of area of the deforming members. The equations were used to quantify the mechanical effect of interlayer offset and to define target property values for the subsequent GAN-CAD-FEA workflow. This analytical stage is essential because it gives the generative design process an interpretable mechanical basis rather than relying only on geometry generation.

### 2.4. Digital Design and Validation Pipeline: GAN-CAD-FEA Integration

The analytical model defines the relationship between lattice geometry and mechanical response, but patient-specific implants require manufacturable three-dimensional geometries rather than equations alone. Therefore, the analytical model was coupled with a digital design and validation workflow combining conditional generative adversarial networks (cGANs), CAD reconstruction, and finite-element analysis (FEA). The objective of this workflow was to solve an inverse design problem: given a target porosity, target modulus, and anatomical design requirement, generate candidate lattice geometries that preserve pore space while achieving the prescribed mechanical response.

The cGAN was conditioned on target porosity P and target modulus E_target. The generator received a random latent vector z and a conditioning vector containing the target design parameters, then produced a three-dimensional voxel representation of a lattice unit cell. The generated samples were not used as independent experimental data; they were used to test whether the analytical stiffness targets could be converted into geometrically valid lattice candidates. The GAN stage therefore served as an inverse-design module within the computational workflow.

The conditioning process can be expressed as:

(11) $$\hat{x}=G\left(z,c\right),\;c=\left[P,E_{target}\right].$$

Wasserstein loss with gradient penalty was used to improve training stability and reduce mode collapse. Synthetic training pairs were generated from the analytical model using porosity values P = {35%, 50%, 65%, 75%, 89%} and interlayer-offset states ranging from K = 0 to K = 0.5. This produced 3000 paired samples linking target mechanical properties to voxel-based lattice geometries.

The nature of this training set defines the scope of what the generator can and cannot do, and that limitation is stated here explicitly. Because the 3000 training pairs were produced from Equations (2)–(10) rather than from experiments or high-fidelity simulations, the cGAN can only reproduce the relationships already embedded in the analytical model. It adds no independent mechanical information, and it cannot represent as-built geometric deviation, melt-pool-driven strut irregularity, surface roughness, residual stress, or any other source of real manufacturing variability. The generator is therefore used here strictly as an inverse geometry-generation module that returns manufacturable voxel candidates consistent with a prescribed target pair (P, E_target_), and not as a predictive surrogate for the mechanical behavior of printed parts. The finite-element step that follows is, correspondingly, a consistency check on the geometry pipeline rather than an independent validation of the underlying physics. Extending the training set with experimental or process-aware data, as has been demonstrated for lattice and triply periodic minimal surface architectures conditioned on process parameters [30,31], is a necessary step before the generative component can be regarded as predictive for as-built components.

Generated voxel lattices were converted into engineering-ready geometries through surface extraction and CAD reconstruction. Marching cubes was used to extract triangulated surfaces from voxel boundaries, followed by smoothing to reduce stair-step artifacts. The reconstructed CAD solids enabled additional design operations that are difficult to impose directly inside the neural generator, including local fixation zones, anatomical avoidance volumes, and graded transitions in structural kurtosis across the implant height.

Virtual mechanical validation was performed using FEA under uniaxial compression, matching the assumptions of the analytical model. The bottom face of each unit cell was fixed, and a uniform displacement corresponding to 0.5% axial strain was applied to the top face. Ti-6Al-4V was modeled as a linear elastic isotropic material with E = 103 GPa and a Poisson’s ratio ν = 0.34. The FEA-derived effective modulus was calculated from the ratio between applied stress and applied strain:

(12) $$E_{FEA}=\frac{\sigma_{applied}}{\varepsilon_{applied}}.$$

The applied stress was obtained from the total reaction force divided by the projected cross-sectional area. The relative error between the FEA result and the analytical target was calculated as:

(13) $$\delta =\frac{\left|E_{FEA}-E_{analytical}\right|}{E_{analytical}}\times 100.$$

**Figure 4:**
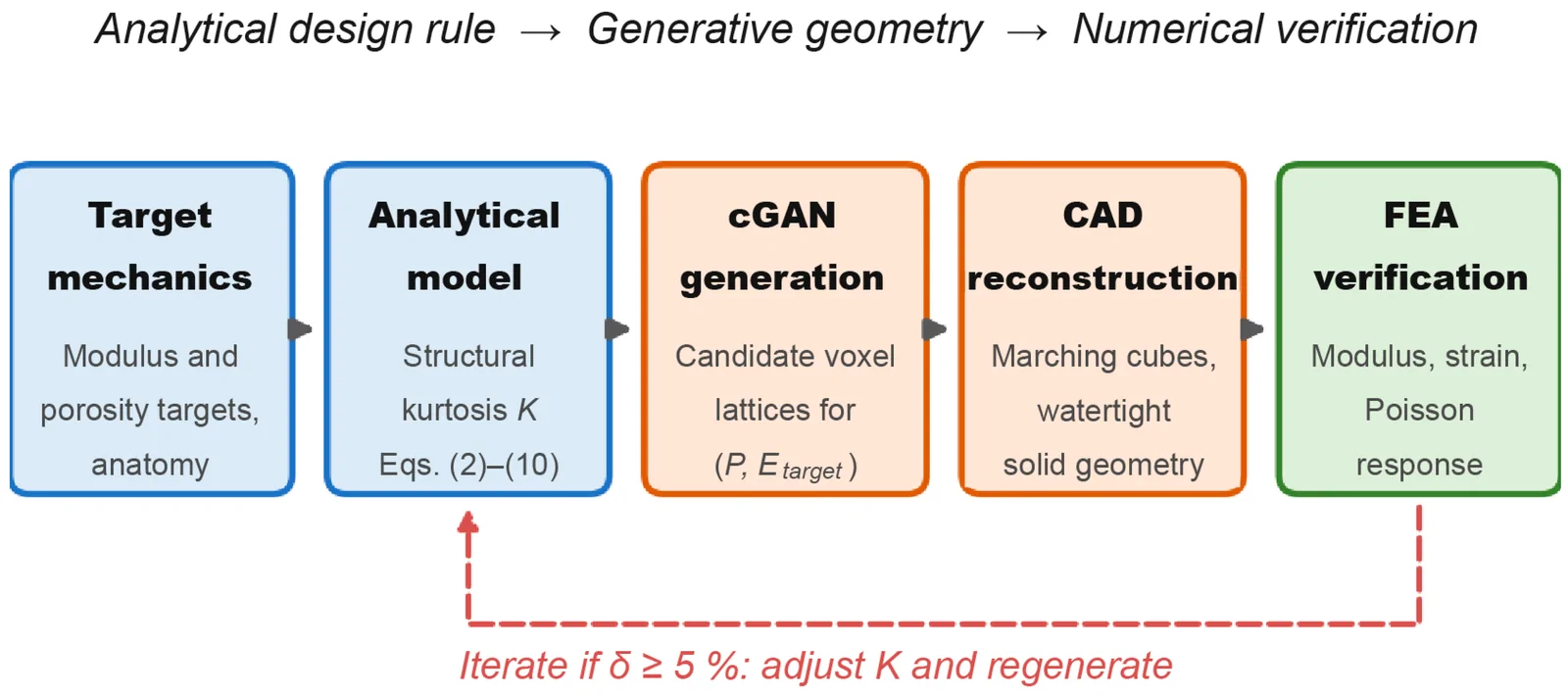
Integrated digital pipeline linking analytical kurtosis-based mechanics with conditional GAN generation, CAD reconstruction, and FEA-based verification, including the iteration loop entered when the verified modulus deviates from the analytical target by 5% or more.

Generated designs with δ < 5% were considered mechanically consistent with the analytical target. The complete workflow is summarized in Figure 4, which shows how analytical property targets were converted into candidate voxel lattices, manufacturable CAD models, and validated FEA outputs.

As shown in Figure 4, the workflow connects theoretical design rules with candidate implant geometry that satisfies the imposed geometric constraints. The analytical model supplies the mechanical target; the cGAN generates design alternatives; CAD reconstruction imposes engineering constraints; and FEA confirms whether the reconstructed lattice preserves the intended stiffness, strain capacity, and lateral response before fabrication.

The reproducibility of this workflow depends on information that is not fully conveyed by the description above, and this is acknowledged. The generator architecture, optimizer settings, learning rate, batch size, number of training epochs, and random seed, together with the finite-element solver, element type, mesh density, and mesh-convergence study, govern whether the reported values can be reproduced independently. These settings, the trained generator weights, the generated voxel datasets, the reconstructed CAD models, and the finite-element input files are reported in full and made available as described in the Data Availability Statement, so that the entire pipeline, and not only its reported outputs, can be re-executed and checked.

### 2.5. Benchmarking and Manufacturing-Sensitivity Analysis

Two additional analyses were performed on the analytical model: one to place the proposed design parameter in a quantitative context against existing lattice families, and one to establish how robust the predicted moduli are to the geometric deviations introduced by additive manufacturing. Both are analytical; neither uses experimental input.

For benchmarking, established lattice families were represented by the Gibson–Ashby relation E*/E_s_ = Cρ^n^, in which the exponent n characterizes the deformation regime: *n* = 1 for stretching-dominated topologies such as the simple cubic and octet-truss lattices, *n* = 2 for bending-dominated topologies such as body-centered-cubic and foam-like architectures, and intermediate values of approximately 1.6 and 1.8 for gyroid and diamond triply periodic minimal surface architectures, respectively [13,17,25,26]. Each family was required to reduce its own effective modulus by the same factor of 6.25 that structural kurtosis provides at fixed porosity, starting from the same relative density (ρ = 0.352; that is P = 65%), and the porosity, normalized strut thickness, and absolute strut thickness at a = 1.2 mm required to do so were computed.

The quantity compared in this way is prefactor-free, and this is what makes the comparison meaningful despite the wide scatter of reported topology-specific coefficients. For any architecture obeying E* = CE_s_ρ^n^, reducing the modulus by a factor F requires reducing the relative density by F^1/n^, and the coefficient C cancels. The comparison is consequently a statement about how much porosity each deformation regime must trade for a given stiffness reduction. It is not a claim about the absolute modulus of any particular topology at 65% porosity, which does depend on C and which this study does not attempt to predict.

For the manufacturing-sensitivity analysis, the derived scaling laws were differentiated with respect to strut thickness. Because E_I_ ∝ (t/a)^2^ and E_II_ ∝ (t/a)^4^, a relative deviation in as-built strut thickness propagates to the effective modulus, as shown in Equation (14):

(14) $$\frac{\Delta E}{E}\approx n_{t}\frac{\Delta t}{t},$$

with n_t_ = 2 for the aligned lattice and n_t_ = 4 for the interlayer-shifted lattice. Deviations were evaluated for nominal strut-thickness errors of ±5% and ±10%, which span the dimensional accuracy commonly reported for thin laser powder-bed fusion features, and for the reduction in effective load-bearing cross-section caused by surface roughness and partially fused powder, modeled as the loss of a 25 µm layer from each free surface of the strut. No measurements were used; the analysis is a propagation of the derived scaling laws and is intended to indicate the magnitude of the deviation to be expected upon fabrication and which of the two configurations is the more process-sensitive.

## 3. Results

### 3.1. Effective Modulus of Rigid and Kurtosis-Controlled Lattices

The effective-modulus analysis indicates that structural kurtosis substantially expands the stiffness design space of porous Ti-6Al-4V lattices. In both lattice configurations, the effective modulus increased as porosity decreased, reflecting the larger solid fraction and greater load-bearing cross-sectional contribution. However, the aligned and interlayer-shifted lattices followed different mechanical regimes. The aligned lattice retained a stretching-dominated response, whereas the kurtosis-controlled lattice exhibited lower effective modulus values because the interlayer offset redirected part of the axial load into bending-dominated deformation.

The calculated modulus values are summarized in Table 2. This table quantifies the central design claim of the study analytically: the same porosity level can be assigned different mechanical functions by changing the deformation mechanism rather than the pore volume. The values should therefore be interpreted as the two mechanical bounds of the kurtosis-controlled lattice family, the aligned K = 0 limit and the shifted compliant limit, both of which follow from Equations (3) and (5) rather than from a fitted relationship.

As shown in Table 2, the most informative comparison occurs at 65% porosity. At this fixed pore-volume condition, the aligned lattice reaches 16.5 GPa, which is suitable for mechanically supportive cortical-like loading. In contrast, the kurtosis-controlled configuration reaches 2.64 GPa, closer to a trabecular-like compliant regime. Thus, structural kurtosis enables mechanical reprogramming without closing pores or reducing the open volume available for biological integration.

**Figure 5:**
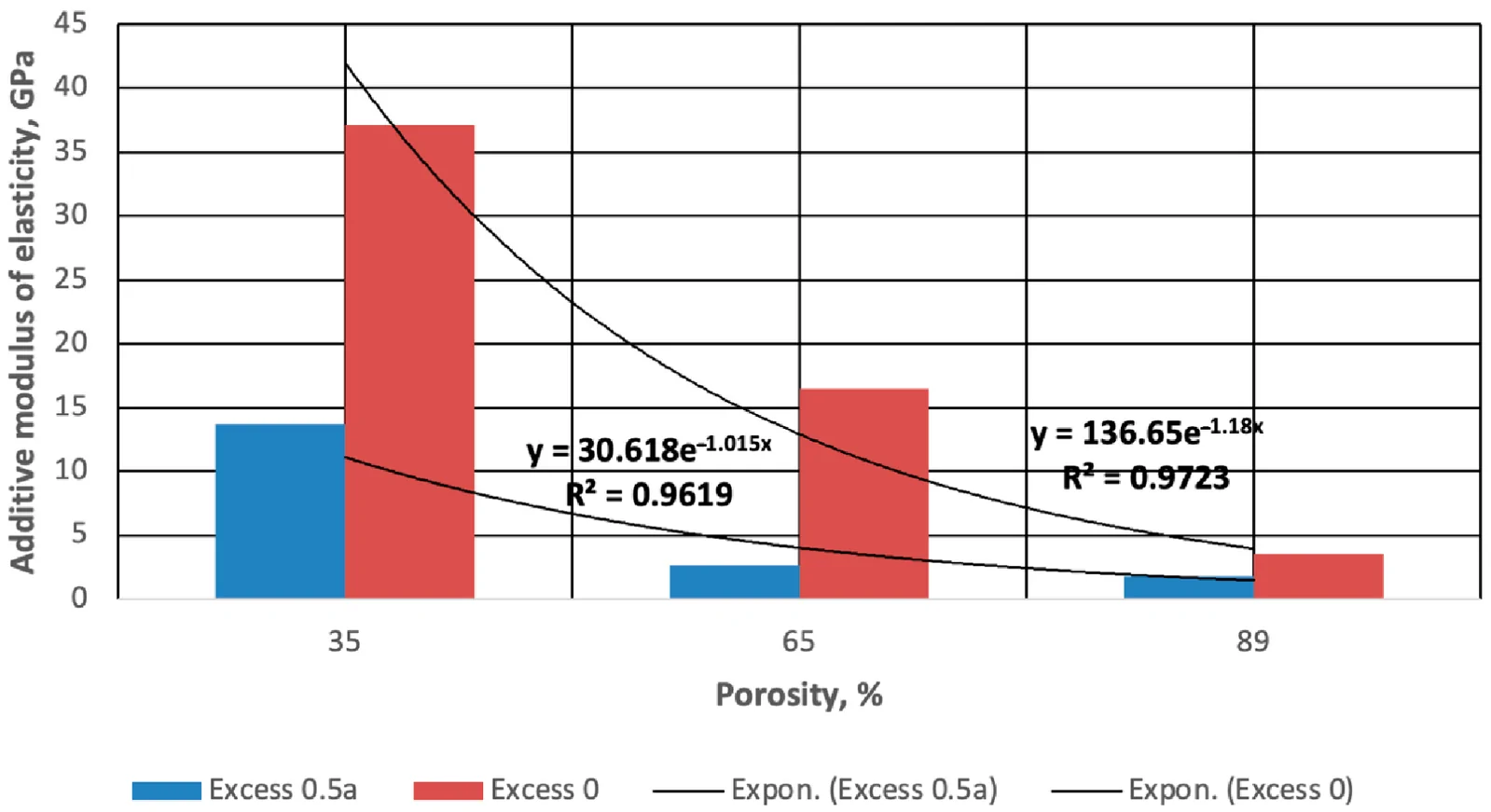
Effective modulus as a function of porosity and interlayer offset. The kurtosis-controlled configuration substantially expands the available stiffness design range.

Figure 5 visualizes this divergence between the two deformation modes. The aligned lattice shows a steep modulus increase as porosity decreases, while the kurtosis-controlled lattice remains in a lower and more compliant stiffness range over the same porosity interval.

The combined interpretation of Table 2 and Figure 5 supports the main design claim of the study: stiffness can be tuned independently from pore-volume requirements. This is important for orthopedic implants because porosity must remain sufficient for osseointegration, whereas stiffness must be adapted to local bone quality and load-transfer requirements.

### 3.2. Elastic Strain Capacity

The elastic strain-capacity results show that structural kurtosis affects recoverable deformation, but that the magnitude and even the sign of the effect depend on which limit governs. The aligned lattice remains at the elastic strain limit of dense Ti-6Al-4V, 0.82%, at every porosity, because its members deform only by axial stretching and compression. For the interlayer-shifted lattice, two bounds must be distinguished: the kinematic upper bound of Equation (8), which considers structural compliance alone, and the local-yield limit of Equation (9), which accounts for the peak outer-fiber strain generated by bending and therefore for the onset of plastic hinging. The latter is the physically admissible value and is reported here as the governing result.

Both bounds are presented in Table 3, together with the ratio of the governing value to the aligned reference. The table makes explicit the difference between the deformation the structure could geometrically accommodate and the deformation it can accommodate while the constituent Ti-6Al-4V remains elastic.

Table 3 shows that the strain benefit of structural kurtosis is confined to the high-porosity regime. At 89% porosity, the shifted lattice tolerates 1.37% recoverable strain against 0.82% for the aligned lattice, an increase of 67%. At 65% porosity, the local-yield limit falls to 0.68%, slightly below the aligned reference, because the struts are no longer slender enough for the compliance gain to offset the local bending stress; the crossover predicted by Equation (9) lies at 74.1% porosity for λ = 1. The values of 20.5% and 5.12% are retained in Table 3 as the kinematic upper bound, but they should not be interpreted as recoverable strains for a metallic lattice, because they would require the entire connecting member to reach the axial elastic limit simultaneously, which the bending stress distribution precludes.

**Figure 6:**
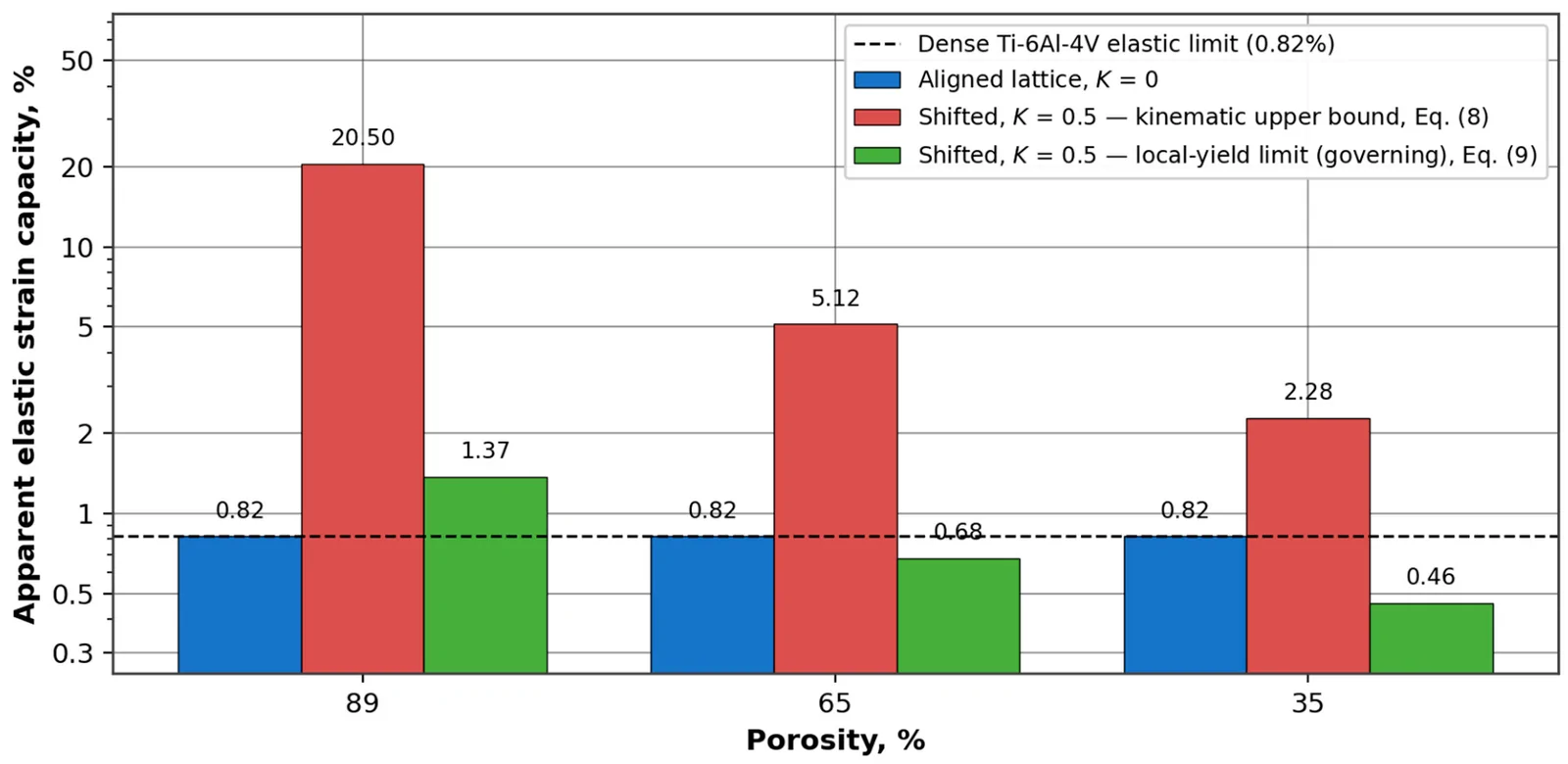
Apparent elastic strain capacity as a function of porosity and interlayer offset, showing the kinematic upper bound of Equation (8) and the governing local-yield limit of Equation (9) against the aligned reference. The strain advantage of the shifted configuration persists only above approximately 74% porosity.

Figure 6 illustrates both bounds on a logarithmic scale, together with the elastic limit of the dense alloy. The difference between the two configurations is not a matter of material stiffness alone; it reflects a different structural deformation mechanism, and the two bounds diverge precisely because that mechanism redistributes stress within the member rather than across it.

The strain-capacity result remains clinically relevant, but its scope is narrower than the compliance-based estimate suggests. Deformation-tolerant behavior can be programmed through interlayer offset in highly porous regions of an implant—for example, in trabecular-contact or metaphyseal zones—whereas at intermediate porosity, the principal benefit of structural kurtosis is stiffness reduction rather than increased recoverable strain. Reported compressive responses of laser powder-bed fusion and electron-beam Ti-6Al-4V lattices are consistent with this picture, showing the onset of yielding at global strains of, at most, a few percent, with bending-dominated architectures entering plastic hinging earlier than stretching-dominated architectures of comparable relative density [21,22,23,25]. Designs that rely on large recoverable deformation should therefore be placed in the high-porosity branch of the design space, and verified experimentally before use.

### 3.3. Poisson Response and Lateral Deformation Control

The predicted Poisson response indicates that interlayer offset also modifies lateral deformation behavior. At a half-cell offset, high-porosity lattices exhibit an auxetic-like response; that is, a negative apparent Poisson ratio. The sign convention is stated explicitly here because it determines the design implication: a negative Poisson ratio means that axial compression is accompanied by lateral contraction rather than by the lateral expansion of a conventional solid, and that axial tension is accompanied by lateral expansion. This behavior was strongest at 89% porosity, where the apparent Poisson ratio was approximately −0.10.

**Figure 7:**
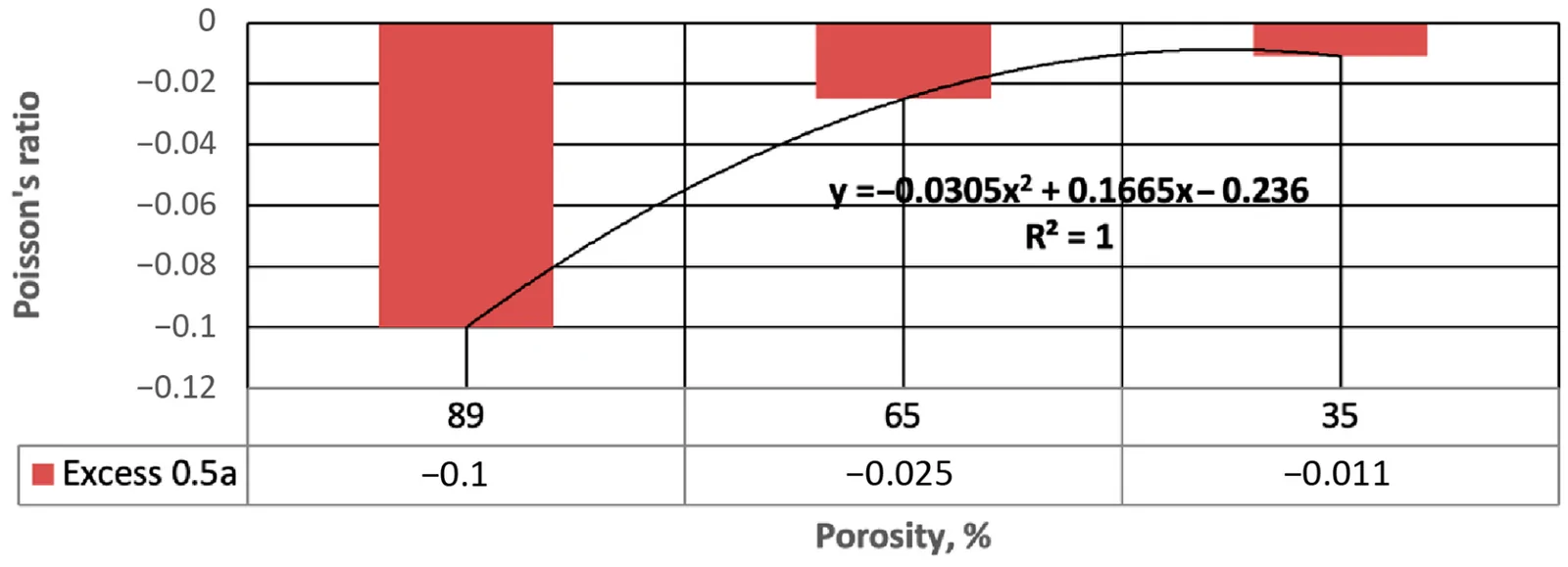
Apparent Poisson ratio as a function of porosity at a half-cell interlayer offset. High-porosity lattices display a stronger auxetic-like response.

Figure 7 shows the relationship between porosity and apparent Poisson ratio for the half-cell interlayer-offset condition.

As shown in Figure 7, the magnitude of the negative Poisson response decreases as porosity decreases, approaching approximately −0.011 at 35% porosity. The lateral response can therefore be moderated through the same geometric parameter that controls axial stiffness. The implant-design consequences follow directly from the sign and are the opposite of those of a conventional lattice. Under physiological compression, the lattice contracts laterally, which lowers the radial pressure exerted on surrounding cancellous bone and reduces hoop stress in a press-fit cavity, but which also removes lateral expansion as a contributor to primary stability. Under a tensile or pull-out load, the same lattice expands laterally and therefore tends to interlock with the surrounding bone, which is the mechanism by which auxetic architectures are usually argued to improve pull-out resistance. The predicted magnitudes are small, between −0.011 and −0.10, and they have not been measured, so these effects should be regarded as second-order modifiers of interface behavior rather than as a primary fixation mechanism.

Structural kurtosis therefore has a dual mechanical function: it controls axial compliance and influences lateral compatibility at the bone–implant interface. This makes it more versatile than porosity alone, which mainly changes the amount of solid material but does not directly program lateral deformation behavior.

### 3.4. GAN-CAD-FEA Digital Validation

The GAN-CAD-FEA workflow was evaluated by comparing analytical mechanical targets with FEA-derived mechanical responses from generated and reconstructed lattice candidates. This validation step tested whether the digital design pipeline preserved the intended mechanical behavior after voxel generation, surface extraction, CAD reconstruction, and numerical compression testing.

Table 4 compares analytical effective modulus values with FEA-derived modulus values for six representative GAN-CAD lattice candidates. The selected cases cover high-, intermediate-, and lower-porosity conditions and include both aligned and shifted configurations.

Five of the six relative errors in Table 4 remain below the adopted 5% validation threshold; scenario A6, the fully shifted configuration at 35% porosity, deviates by 6.5% and is reported as such. The shifted compliant configurations show consistently larger deviations than the aligned configurations, which is expected because bending-dominated lattices are more sensitive to local curvature, surface smoothing, strut-thickness variation, and stress concentration introduced during CAD reconstruction.

**Figure 8:**
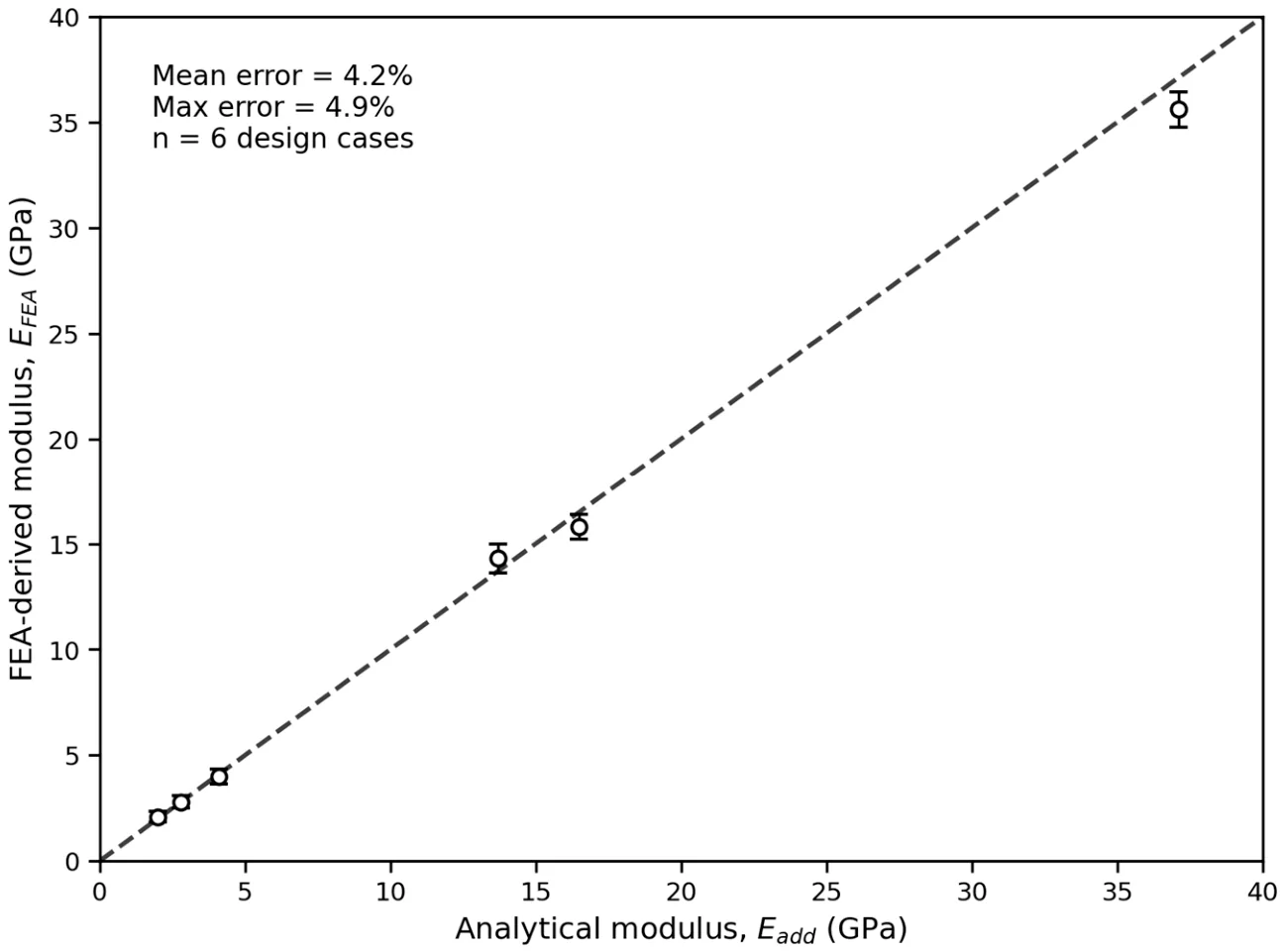
Correlation between analytical effective modulus (E_add_) and FEA-derived effective modulus (E_FEA_) for representative GAN-CAD lattice candidates. Here, E_add_ denotes the effective modulus predicted by the analytical model, E_FEA_ denotes the effective modulus obtained from finite element analysis, GPa denotes gigapascals, and n denotes the number of evaluated design cases (n = 6). The dashed line indicates ideal one-to-one agreement.

The agreement between analytical and FEA-derived moduli is further shown in Figure 8. The data points follow the one-to-one reference line closely, indicating that the generated and reconstructed lattice candidates retained the mechanical targets imposed by the analytical model.

Figure 8 confirms that the analytical model can be translated into digital geometries with controlled and quantifiable deviations. This is important because generative models can produce visually plausible structures that do not necessarily preserve mechanical intent. In the proposed workflow, the analytical model constrains the target behavior, while FEA provides a verification step. The scope of that verification should be stated precisely: it covers six design cases, it uses the same idealized geometry and the same linear elastic isotropic material model as the analytical derivation, and the generator that produced the candidates was trained on data generated by that derivation. Five of the six scenarios fall below the adopted 5% threshold; the sixth, A6 at 35% porosity and K = 0.5, deviates by 6.5%, which is reported here rather than absorbed into the threshold. Table 4 therefore establishes that the digital pipeline preserves the intended geometry and the intended stiffness through voxel generation, surface extraction, CAD reconstruction, and meshing to within about 7%. It does not establish that either the model or the pipeline reproduces the behavior of a physically printed lattice, which would require fabrication and mechanical testing.

**Figure 9:**
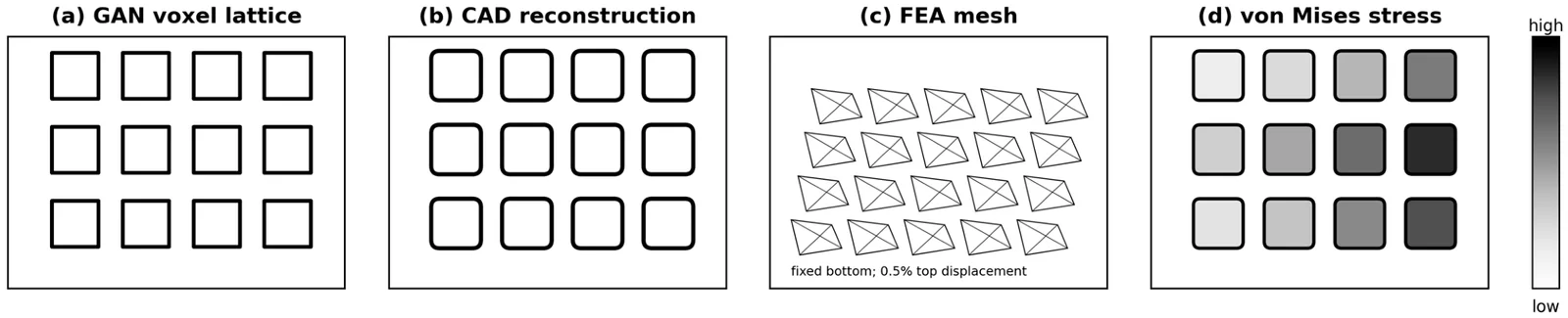
Representative digital validation sequence for a kurtosis-controlled lattice: (**a**) GAN-generated voxel lattice, (**b**) CAD reconstruction, (**c**) FEA mesh and boundary conditions, and (**d**) von Mises stress distribution under compression.

A representative digital validation sequence is shown in Figure 9. The figure illustrates how a generated lattice candidate is converted into a watertight CAD model that can be meshed and analyzed.

As shown in Figure 9, the cGAN generates the initial voxel-based topology, CAD reconstruction converts the voxel field into a smooth solid geometry, FEA meshing enables numerical compression testing, and the stress map identifies local stress concentrations. This sequence demonstrates that structural-kurtosis parameterization can move from equation-level design to digital geometry while preserving the intended stiffness response.

The validation was extended to elastic strain capacity and apparent Poisson response for selected GAN-CAD models. These results are summarized in Table 5.

The results in Table 5 show that the FEA-derived strain capacity and apparent Poisson response follow the analytical predictions, with two qualifications that are stated here rather than left to the reader. First, the strain values in Table 5 correspond to the kinematic bound of Equation (8): the finite-element model is linear elastic and contains no yield criterion, so it necessarily reproduces the compliance-based strain and cannot indicate the onset of local yielding. The governing recoverable strains are those of Equation (9), reported in Table 3. Second, the finite-element and analytical Poisson ratios agree in sign and in order of magnitude but not in value: −0.07 against −0.10 at 89% porosity and −0.04 against −0.025 at 65% porosity. Smoothing of the offset nodes during CAD reconstruction reduces the geometric re-entrancy that produces the effect, which is the most likely explanation, but the discrepancy has not been resolved and the absolute values of the apparent Poisson ratio should be treated as uncertain. Subject to these qualifications, the high-porosity shifted model preserved both high geometric compliance and a negative lateral response, while the aligned high-porosity model remained close to the base elastic strain limit.

### 3.5. Patient-Specific Design Scenarios

The analytical and digital results suggest two patient-specific design strategies. These are presented as conceptual scenarios and not as validated designs: no anatomical geometry was segmented from patient imaging, no bone-implant construct was simulated, and no implant was fabricated. First, a single implant can be assigned a stiffness gradient along the bone–implant interface by varying the interlayer offset while maintaining fixed porosity. This allows different regions of the implant to be tuned for cortical support, trabecular compliance, or transitional load sharing without changing the overall pore-volume design. Second, semi-rigid reconstruction elements can be designed with controlled deformation zones that allow limited mobility within a target angular range.

**Figure 10:**
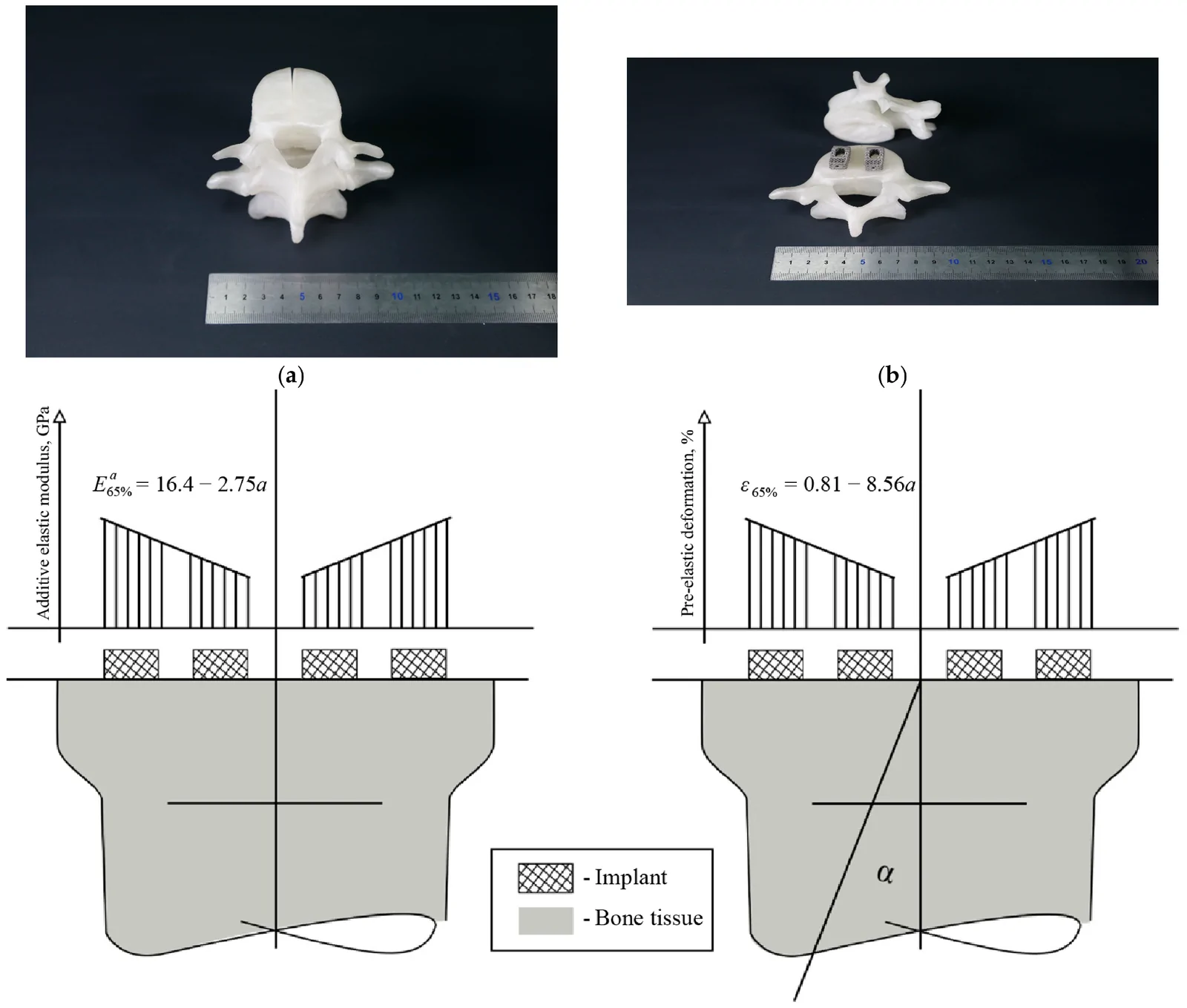
Conceptual patient-specific implant strategies based on structural kurtosis: (**a**) vertebral model, (**b**) porous titanium lattice elements inserted into a vertebral model, and (left, right) graded implant strategies for stiffness grading and controlled angular mobility.

These concepts are illustrated in Figure 10 using vertebral implant scenarios and schematic graded-interface designs.

As shown in Figure 10, structural kurtosis can be applied as a spatially variable design parameter rather than as a single uniform value. This allows one implant to combine stiff fixation regions with more compliant deformation zones. Such grading may be useful in spinal or joint-related applications where excessive stiffness can increase stress shielding, while excessive compliance can compromise stability.

**Figure 11:**
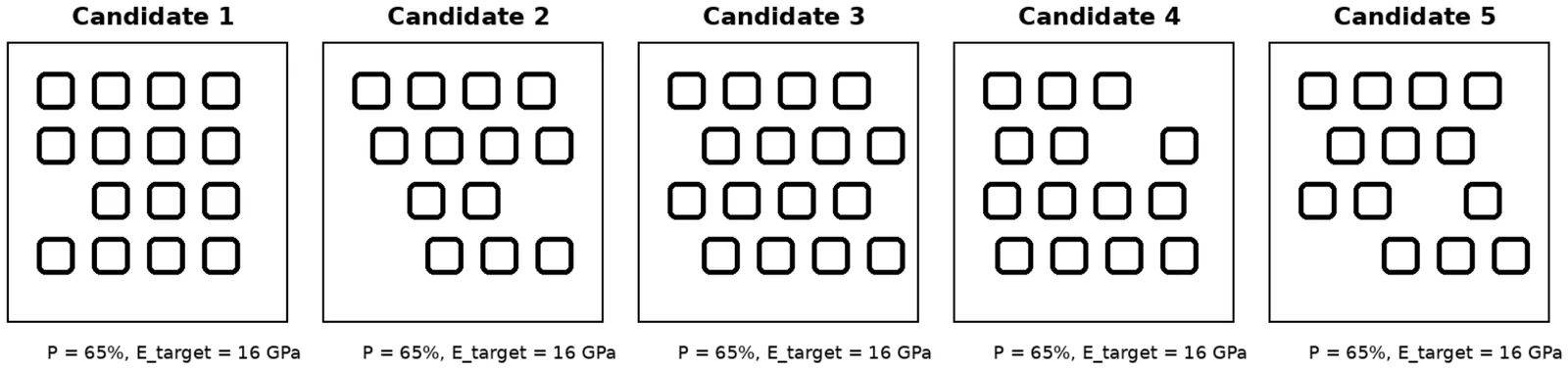
Diversity of GAN-generated lattice candidates for identical target properties (P = 65%, E_target = 16 GPa). This design multiplicity supports anatomical and manufacturing optimization without changing the mechanical target.

The generative component further expands the design space by producing multiple geometrically distinct candidates for the same target porosity and modulus. This design multiplicity is shown in Figure 11.

Figure 11 demonstrates that a single mechanical target does not correspond to only one possible lattice geometry. Instead, the GAN-CAD workflow provides alternative designs with equivalent target properties. This gives the designer freedom to select an implant architecture according to anatomical fit, support-removal feasibility, expected osseointegration surface, local stress distribution, and manufacturability. The scenarios in Figure 10 and Figure 11 should be read as illustrations of how the parameter could be deployed in a patient-specific workflow, and not as demonstrated patient-specific designs; establishing the latter requires image-based anatomical modeling, fabrication, and mechanical testing, none of which was undertaken here.

### 3.6. Benchmarking Against Established Lattice Topologies

To establish whether structural kurtosis offers a genuine advantage over existing design routes, rather than an alternative description of the same trade-off, the kurtosis-controlled lattice was compared quantitatively with established topology families using the procedure outlined in Section 2.5. The comparison was framed as a design question: starting from the same reference state (P = 65%, E = 16.48 GPa), what must each architecture give up in order to reach an effective modulus of 2.64 GPa?

**Figure 12:**
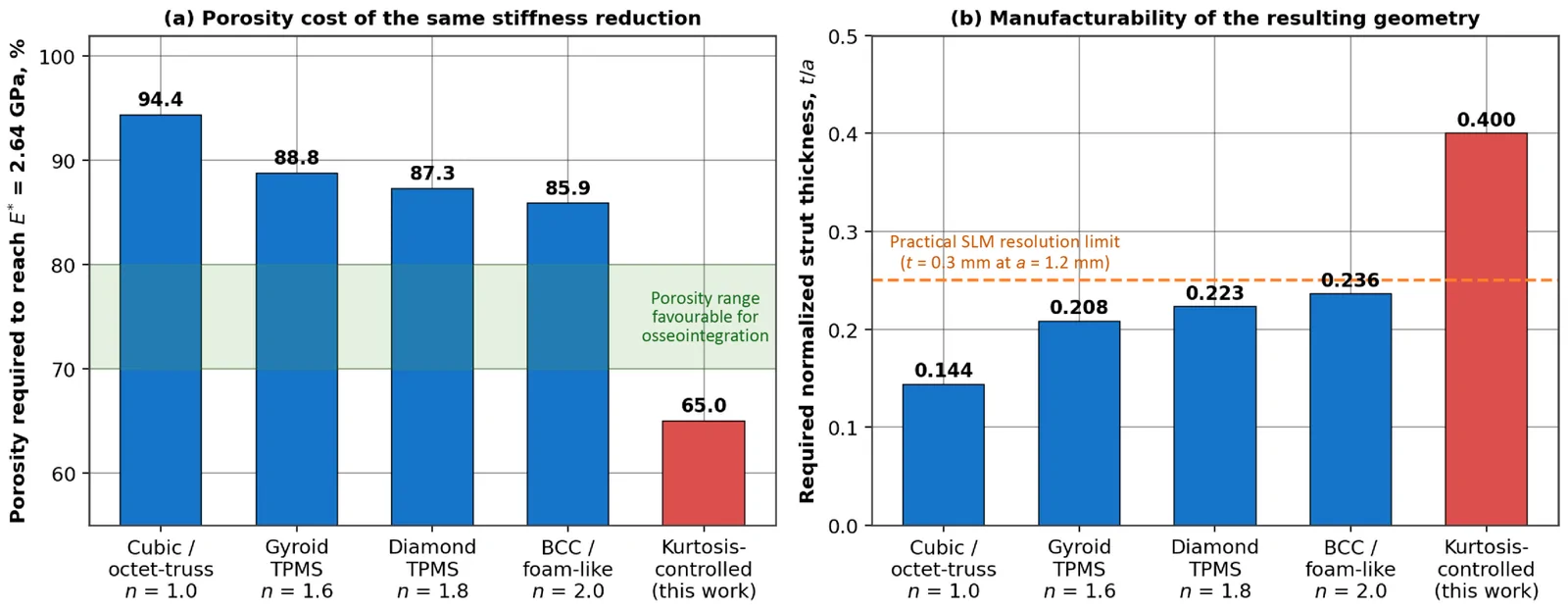
Quantitative benchmarking of the kurtosis-controlled lattice against established topology families. (**a**) Porosity required for each architecture to achieve, through porosity increase alone, the same 6.25-fold modulus reduction provided by structural kurtosis at constant porosity. Blue bars represent the established reference lattice topologies, while the red bar represents the proposed kurtosis-controlled lattice. The green shaded region indicates the porosity range considered favorable for osseointegration. (**b**) Corresponding normalized strut thickness required for each architecture. The orange dashed line indicates the practical SLM resolution limit for load-bearing features.

The results are summarized in Table 6 and Figure 12.

**Table 6:** Quantitative benchmarking of the kurtosis-controlled lattice against established topology families. Each family is required to reach E* = 2.64 GPa from the same reference state (P = 65%, E = 16.48 GPa) using its own Gibson–Ashby exponent n. Strut thicknesses are given for a 1.2 mm unit cell.

Lattice Architecture	Deformation Regime (Exponent n)	Porosity Required for a 6.25-Fold Modulus Reduction (%)	Required t/a	Strut Thickness at a = 1.2 mm (mm)	Stiffness Range at Fixed P = 65%	Limiting Factor
Simple cubic/octet-truss	Stretching-dominated (*n* = 1.0)	94.4	0.144	0.17	Single value (1.0×)	Below practical SLM feature resolution; very low strength
Gyroid TPMS	Intermediate (n ≈ 1.6)	88.8	0.208	0.25	Single value (1.0×)	At SLM resolution limit; porosity above ingrowth window
Diamond TPMS	Intermediate (n ≈ 1.8)	87.3	0.223	0.27	Single value (1.0×)	At SLM resolution limit; porosity above ingrowth window
BCC/foam-like	Bending-dominated (*n* = 2.0)	85.9	0.236	0.28	Single value (1.0×)	At SLM resolution limit; low strength
Kurtosis-controlled cubic (this work)	Tunable, *n* = 1 → 2 via K	65.0 (unchanged)	0.400	0.48	Factor 6.25 (K = 0 → 0.5)	None within the studied range; sensitivity to strut deviation (Table 7)

**Table 7:** Analytical sensitivity of the predicted effective moduli to as-built strut-thickness deviation, obtained by propagating the deviation through Equations (3) and (5) using Equation (14). The roughness cases model the loss of a 25 µm layer from each free surface of the strut.

Source of Deviation	Δt/t (%)	ΔE_I/E_I, Aligned Lattice (%)	ΔE_II/E_II, Shifted Lattice (%)
Nominal as-designed geometry	0	0	0
Strut oversizing, adhered partially fused powder	+5	+10.3	+21.6
Strut oversizing, upper case	+10	+21.0	+46.4
Strut undersizing, incomplete fusion	−5	−9.8	−18.5
Strut undersizing, upper case	−10	−19.0	−34.4
Surface roughness R_a ≈ 25 µm, t = 0.48 mm (a = 1.2 mm)	−10.4	−19.7	−35.6
Surface roughness R_a ≈ 25 µm, t = 0.24 mm (a = 0.6 mm)	−20.8	−37.3	−60.7

Table 6 shows that no established topology reaches the target stiffness at 65% porosity. Stretching-dominated architectures such as the simple cubic and octet-truss families, for which *n* = 1, must be thinned to 94.4% porosity, and triply periodic minimal surface and body-centered-cubic architectures, with exponents between 1.6 and 2.0, to between 85.9% and 88.8%. In every case, the required porosity lies above the 70–80% window usually associated with favorable bone ingrowth combined with mechanical continuity, and the corresponding strut thickness falls to between 0.17 and 0.28 mm at a 1.2 mm cell, at or below the practical resolution of selective laser melting for load-bearing features. The kurtosis-controlled lattice reaches the same modulus at 65% porosity with 0.48 mm struts, because the reduction is obtained by changing the deformation regime rather than by removing material.

The penultimate column of Table 6 expresses the same result as a design range. At a fixed porosity of 65%, each established topology delivers a single effective modulus, whereas varying K from 0 to 0.5 spans a factor of 6.25 about that value, with no change in pore volume and no change in unit-cell family. This is the quantitative advantage claimed for the parameter. It is not that a lower absolute stiffness can be reached, since adding porosity can also deliver that, but that the stiffness is decoupled from the pore architecture and from the minimum manufacturable feature size.

The comparison also identifies where kurtosis control is not advantageous, and this limit should be stated with the same clarity. As shown in Section 3.2, the recoverable elastic strain of the shifted lattice exceeds that of the aligned lattice only above 74% porosity, so bending-dominated established topologies such as body-centered-cubic remain competitive when large recoverable deformation, rather than stiffness at fixed porosity, is the governing design requirement. Table 6 should therefore be read as delimiting the region of the design space in which structural kurtosis is the more efficient parameter, not as a claim of general superiority.

### 3.7. Sensitivity of the Predicted Moduli to As-Built Manufacturing Deviations

Because no specimens were fabricated in this study, the influence of selective laser melting on the predicted properties was assessed analytically by propagating strut-thickness deviations through the scaling laws derived in Section 2.3 using the procedure outlined in Section 2.5. This analysis does not replace experimental characterization. Its purpose is to indicate the magnitude of the deviation that should be expected when the present designs are printed, and to identify which of the two configurations is the more process-sensitive.

**Figure 13:**
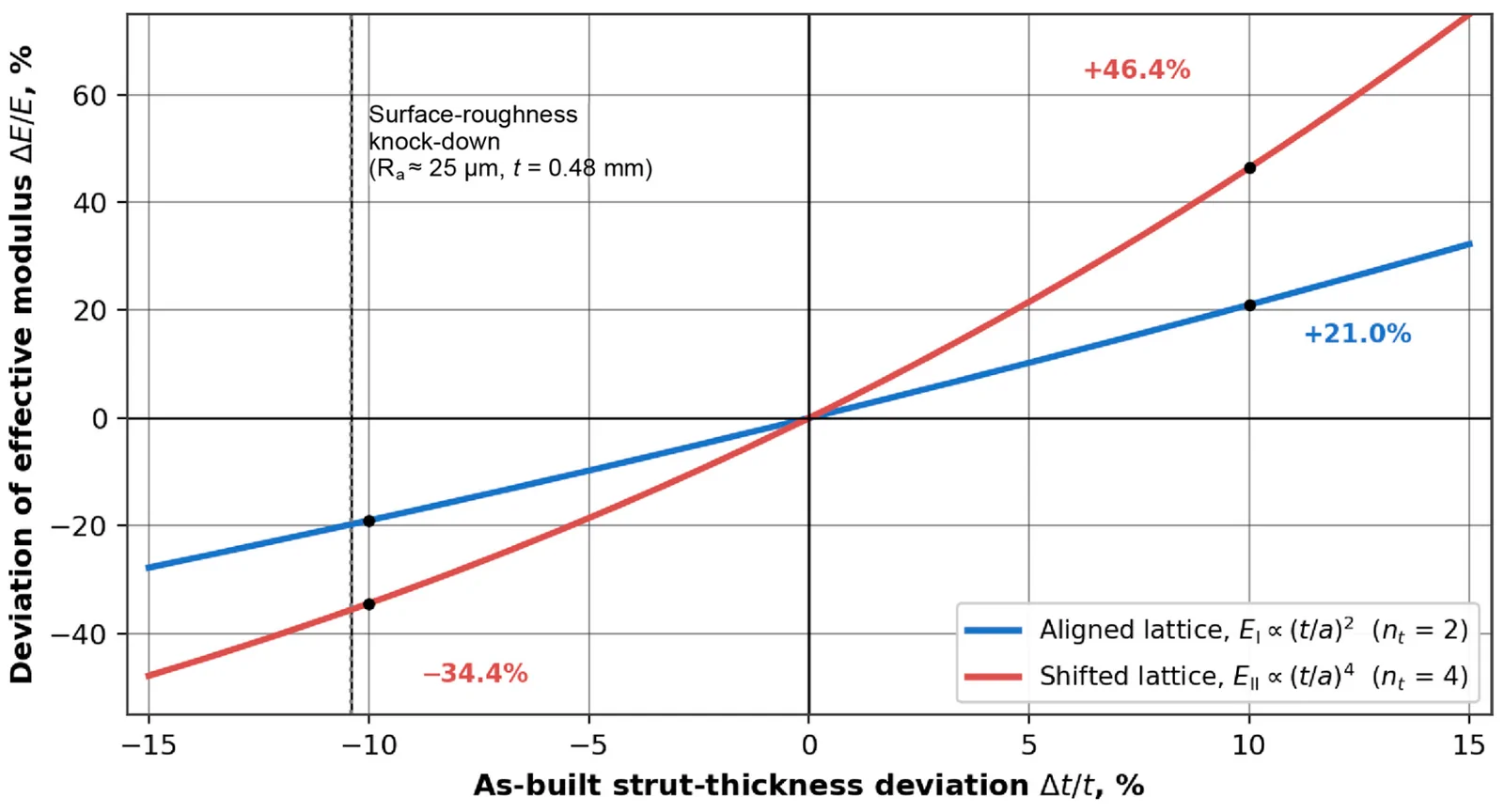
Propagation of as-built strut-thickness deviation to the effective modulus for the aligned (n_t = 2) and interlayer-shifted (n_t = 4) configurations, with the surface-roughness knock-down case for a 0.48 mm strut indicated.

The results are given in Table 7 and Figure 13.

The exponent that gives the kurtosis-controlled lattice its wide stiffness range is also what makes it sensitive to manufacture. A 10% strut-thickness deviation, which lies within the range commonly reported for thin laser powder-bed fusion features, changes the aligned modulus by 21% but the shifted modulus by 46%. Modeling the loss of load-bearing capacity caused by surface roughness and partially fused powder as a 25 µm reduction from each free surface of a 0.48 mm strut gives an effective thickness deficit of 10.4%, and predicts modulus reductions of 19.7% and 35.6% for the aligned and shifted configurations, respectively. The same absolute roughness applied to a 0.24 mm strut, corresponding to a 0.6 mm cell at the same porosity, produces a 20.8% thickness deficit and a 60.7% reduction in the shifted modulus, which shows that the sensitivity grows rapidly as the cell size is reduced.

Two practical consequences follow. First, the kurtosis-controlled design space should be realized at the larger end of the manufacturable cell-size range, where the absolute roughness represents a smaller fraction of the strut section, and post-processing routes that recover a smooth load-bearing cross-section, such as electropolishing [29], are likely to be necessary if the predicted moduli are to be met. Second, the deviations in Table 7 are considerably larger than the 5% agreement obtained between the analytical model and FEA in Table 4. This underlines that the numerical agreement reported in Section 3.4 verifies the digital pipeline and is not an estimate of the accuracy with which a printed component would reproduce the target modulus.

## 4. Discussion

This study shows analytically that interlayer offset can tune the mechanical response of porous Ti-6Al-4V lattices independently of global porosity. This separation is important because porosity and stiffness impose competing requirements in orthopedic implants. Porosity is biologically valuable because it supports fluid transport, tissue ingrowth, vascular access, and surface area for osseointegration. At the same time, porosity is mechanically costly because it reduces load-bearing capacity and can weaken the structure. Treating porosity as the main stiffness-control variable therefore creates a design conflict: improving biological openness often reduces mechanical support. Structural kurtosis addresses this conflict by changing the load path inside the lattice while keeping the pore-volume condition fixed.

The 65% porosity case captures this principle most clearly. At the same pore-volume fraction, the aligned lattice produces a modulus of 16.5 GPa, whereas the kurtosis-controlled lattice produces 2.64 GPa. The magnitude of this modulus shift indicates that interlayer offset changes the dominant deformation mechanism rather than producing only a minor geometric correction. The same porous architecture can behave as a support-oriented structure or as a compliant load-sharing structure depending on interlayer offset. This ability is particularly useful for orthopedic reconstruction because bone is not mechanically uniform. Cortical shells, trabecular cores, metaphyseal regions, and spinal interfaces impose different stiffness demands within relatively small anatomical distances.

The benchmarking in Section 3.6 shows that this stiffness shift cannot be reproduced by established topologies without leaving the intended design window. Obtaining the same 6.25-fold reduction by increased porosity alone requires 94.4% porosity for a stretching-dominated architecture and 85.9% for a bending-dominated one; in both cases the resulting struts measure between 0.17 and 0.28 mm at a 1.2 mm cell, which places the geometry at or below the practical resolution of selective laser melting for load-bearing features and moves the pore architecture outside the porosity range usually associated with reliable bone ingrowth. The distinctive property of structural kurtosis is therefore not that it reaches a lower stiffness, which adding porosity can also achieve, but that it decouples the stiffness from both the pore volume and the minimum manufacturable feature size. Recent comparative studies of body-centered-cubic, triply periodic minimal surface, and auxetic Ti-6Al-4V lattices report broad but density-coupled modulus ranges [25,26,27,28], which is precisely the coupling that the present parameter is intended to break.

The effect of structural kurtosis on apparent elastic strain capacity requires a more careful statement than a compliance argument alone permits. The base alloy defines the local material limit, and the shifted lattice does distribute global deformation through bending and rotation of its members. However, in a bending-dominated member the stress concentrates at the outer fiber of the built-in ends, so yielding and plastic hinging begin there well before the compliance-based estimate of Equation (8) is reached. The local-yield analysis of Section 3.2 therefore replaces the figure of 20.5% at 89% porosity with 1.37%, and shows that at 65% porosity the shifted lattice is in fact marginally less tolerant of recoverable strain than the aligned one. This is consistent with the experimental literature on metallic lattices: compression and fatigue tests on laser powder-bed fusion and electron-beam Ti-6Al-4V lattices report yielding at global strains of at most a few percent, with bending-dominated architectures entering plastic hinging earlier than stretching-dominated architectures of comparable relative density, and with as-built defects and surface notches lowering the onset further [21,22,23,25]. Recoverable strains of the order of 20% are not observed in this alloy system, and the revised estimate brings the model into the range that experiments on comparable metallic lattices support. The clinical argument is unchanged in substance but narrower in scope: many implants fail because the structure is too stiff rather than because the alloy lacks strength, and a kurtosis-controlled lattice addresses that stiffness problem directly, while its usefulness as a deformation-tolerant architecture is restricted to the high-porosity branch of the design space.

The negative Poisson response predicted at high porosity adds a further design variable, but its interpretation must be stated carefully. A negative Poisson ratio means that the lattice contracts, not expands, in the transverse direction when it is compressed axially; lateral expansion occurs under axial tension. For an implant, this reverses the usual argument. Under physiological compression, an auxetic lattice presses less hard against the surrounding cancellous bone, which lowers hoop stress in a press-fit cavity but also removes lateral expansion as a contributor to primary stability; under a tensile or pull-out load it expands laterally and tends to interlock, which is the mechanism by which auxetic architectures are usually argued to improve pull-out resistance. Because the predicted magnitudes are small, from −0.011 at 35% porosity to −0.10 at 89%, and because the finite-element model returns smaller magnitudes still, the effect should be treated as a second-order modifier of interface behavior rather than as a fixation mechanism in its own right. It has not been measured. Structural kurtosis nevertheless provides a tunable lateral-response mechanism in addition to axial stiffness control.

The GAN-CAD-FEA workflow extends the analytical model from property prediction to geometry generation and validation. Analytical equations are transparent and interpretable, but they do not directly generate patient-specific implant geometry. Generative design can produce many shapes, but without a governing mechanical descriptor it may generate structures whose behavior is difficult to explain or validate. The GAN-CAD-FEA pipeline links these strengths: structural kurtosis provides the interpretable design variable, the cGAN produces candidate topologies, CAD reconstruction converts voxel outputs into watertight CAD solids, and FEA verifies whether stiffness, strain capacity, and lateral response remain within the target range. The limits of this arrangement should be stated plainly. The generator was trained exclusively on synthetic pairs derived from Equations (2)–(10), so it reproduces the analytical relationships by construction and cannot extrapolate beyond them. The sub-5% agreement between analytical and FEA moduli in Table 4 therefore demonstrates that the voxel-to-CAD-to-mesh pipeline preserves the intended geometry; it does not constitute independent validation of the underlying mechanics, and it covers only six design cases. Training on experimental or high-fidelity simulation data, and conditioning the generator on process parameters—as has been demonstrated for lattice and triply periodic minimal surface architectures [30,31]—would be required before the generative component could be regarded as predictive for as-built parts.

From a manufacturing perspective, the proposed strategy is attractive because it avoids abrupt switching between unrelated unit-cell families. A single lattice family can be locally shifted to create mechanical gradients. This is advantageous for selective laser melting because it reduces discontinuities, improves geometric continuity, and simplifies design standardization. It also supports sustainable personalized implant production: the mechanical response is programmed through geometry rather than through unnecessary material addition, over-densification, or multiple implant families.

That manufacturing argument must nevertheless be qualified by the sensitivity analysis of Section 3.7. The fourth-power dependence that gives the shifted configuration its wide stiffness range also doubles its sensitivity to strut-thickness error relative to the aligned configuration: a 10% deviation shifts the modulus by 46% rather than 21%, and a realistic roughness allowance of 25 µm per free surface on a 0.48 mm strut already predicts a 36% reduction. Reported characterizations of as-built laser powder-bed fusion Ti-6Al-4V lattices document exactly these sources of deviation, including strut-thickness offset relative to the CAD model, surface texture, adhered partially fused powder, and the build-orientation dependence of the resulting properties [21,22,23,29]. None of these effects are represented in the present analytical model or in the FEA, which assumes ideal geometry and a linear elastic isotropic solid. The practical implication is threefold: the absolute moduli reported here should be treated as design targets for an idealized geometry rather than as predictions for a printed part; the kurtosis-controlled design space is best realized at larger cell sizes, where the roughness allowance represents a smaller fraction of the section; and process-aware calibration, whether through micro-CT-based geometry correction or through post-processing, will be required before the predicted values can be met in practice.

The workflow can be translated into a patient-specific design sequence. A future clinical workflow could begin with CT-based bone assessment, assign regional stiffness targets according to cortical or trabecular quality, generate a kurtosis field across the implant, reconstruct the geometry in CAD, and validate the final design by FEA before fabrication. In this sense, structural kurtosis can act as a bridge between patient-specific imaging, mechanical design, additive manufacturing, and virtual preoperative testing.

### Limitations and Scope of the Present Study

The scope of the present work should be stated without qualification: it is a computational study. All values reported in this manuscript are analytical predictions or finite-element results obtained from idealized geometry. No specimens were fabricated by selective laser melting, no micro-CT imaging was performed, and no compression, fatigue, corrosion, or biological testing was carried out. The manuscript therefore establishes a computational design framework and does not demonstrate a validated design methodology. The title, abstract, results, and conclusions have been framed in these terms, and the claims should be read accordingly.

Within that scope, several specific limitations apply. The analytical model assumes an idealized cubic lattice, linear elasticity, a guided-end idealization of the connecting members with an effective bending span λ that is set to unity in the base case, simplified boundary conditions, and perfectly manufactured struts. The FEA validation covers six design cases and uses the same idealized geometry and material model as the analytical derivation, so the agreement reported in Table 4 is a verification of internal consistency rather than an independent test. The cGAN was trained only on synthetic analytical targets and therefore inherits every assumption of that model. The auxetic-like Poisson response was obtained within the same idealized framework and has not been measured. Additive-manufacturing effects such as strut-thickness deviation, surface roughness, partially fused powder, residual stress, anisotropy, and post-processing will shift the absolute values, and Section 3.7 indicates that the shift may reach several tens of percent for the shifted configuration.

Future work should therefore proceed in the order that these limitations imply: fabrication of the six benchmark configurations by selective laser melting; micro-CT verification of the as-built geometry against the CAD model; quasi-static compression and fatigue testing to measure the effective modulus and the true onset of yielding, against which Equations (5) and (9) can be assessed; corrosion assessment; and biological evaluation of osseointegration. Retraining the generative component on the resulting experimental data would then convert the present inverse-design module into a process-aware one. Subject to that program, the analytical trend identified here is clear and internally consistent: interlayer offset provides a controllable route for tuning lattice stiffness at fixed porosity.

## 5. Conclusions

This study introduced structural kurtosis, defined as the normalized interlayer offset between neighboring lattice layers, as a geometric design parameter for porous Ti-6Al-4V orthopedic implants, and developed it as a computational design framework. The governing expressions were derived from the load-bearing area fraction in the aligned configuration and from Euler–Bernoulli beam theory for guided-end connecting members in the shifted configuration, reproducing the classical Gibson–Ashby exponents *n* = 1 and *n* = 2 without recourse to curve fitting. The parameter enables stiffness, recoverable deformation, and lateral response to be tuned without changing global porosity.

At 65% porosity, the analytical model shifted the effective modulus from 16.5 GPa in the aligned lattice to 2.64 GPa in the kurtosis-controlled lattice, a factor of 6.25 at unchanged pore volume, with intermediate offsets described by a calibrated one-parameter interpolation between these two derived bounds. The recoverable elastic strain capacity of the shifted configuration, evaluated from the peak bending curvature so that local yielding and plastic hinging are accounted for, is 1.37% at 89% porosity and 0.68% at 65% porosity; the compliance-based values of 20.5% and 5.12% are kinematic upper bounds that a metallic lattice cannot realize. The strain advantage of the shifted configuration is confined to porosities above approximately 74%. At high porosity, the shifted configuration also produced an auxetic-like lateral response within the same analytical framework.

A prefactor-free benchmark against established topology families showed that obtaining the same 6.25-fold reduction by increased porosity alone would require between 85.9% and 94.4% porosity and strut thicknesses of 0.17 to 0.28 mm at a 1.2 mm cell, outside both the porosity window favorable to osseointegration and the practical resolution of selective laser melting. A sensitivity analysis showed that the same fourth-power scaling that widens the design space makes the shifted configuration twice as sensitive to as-built strut-thickness deviation as the aligned one, with a 10% thickness error producing a 46% modulus error.

The GAN-CAD-FEA workflow demonstrated that the analytical design rules can be converted into digital lattice candidates, with relative errors below 5% in five of six design cases and 6.5% in the sixth. Because the generator was trained on synthetic data derived from the analytical model, this agreement verifies the geometry pipeline rather than the underlying mechanics.

Overall, structural kurtosis provides a compact and interpretable route for designing biomechanically adaptive Ti-6Al-4V lattice implants, and the present work establishes that route computationally. It does not yet demonstrate it experimentally. The necessary next step is fabrication of the benchmark configurations by selective laser melting, followed by micro-CT verification of the as-built geometry, compression and fatigue testing, and integration into patient-specific CAD planning workflows.

## Figures and Tables

**Table 1:** Strut thickness as a function of porosity and unit-cell size.

m	Porosity, P (%)	1.5 mm Cell	1.2 mm Cell	0.9 mm Cell	0.6 mm Cell	0.3 mm Cell
0.1	89	0.30	0.24	0.18	0.12	0.06
0.2	65	0.60	0.48	0.36	0.24	0.12
0.3	35	0.90	0.72	0.54	0.36	0.18
0.4	10.4	1.20	0.96	0.72	0.48	0.24
0.5	0	1.50	1.20	0.90	0.60	0.30

**Table 2:** Effective elastic modulus of the rigid and kurtosis-controlled Ti-6Al-4V lattice models.

No.	m	Porosity, P (%)	E_I Rigid Lattice (GPa)	E_II Kurtosis-Controlled Lattice (GPa)
1	0.1	89	4.12	0.16
2	0.2	65	16.5	2.64
3	0.3	35	37.1	13.35
4	0.4	10.4	66.0	42.2
5	0.5	0	103.0	103.0

**Table 3:** Elastic strain capacity of the rigid and kurtosis-controlled Ti-6Al-4V lattice models, reported as the kinematic upper bound of Equation (8) and the governing local-yield limit of Equation (9). The local-yield limit is evaluated for the base case λ = 1. At m = 0.5, the lattice is fully dense and the beam idealization does not apply.

No.	m	Porosity, P (%)	ε_I Aligned Lattice (%)	ε_II Kinematic Upper Bound, Equation (8) (%)	ε_II Local-Yield Limit, Equation (9) (%)	Governing ε_II/ε_I
1	0.1	89	0.82	20.5	1.37	1.67
2	0.2	65	0.82	5.12	0.68	0.83
3	0.3	35	0.82	2.28	0.46	0.56
4	0.4	10.4	0.82	1.28	0.34	0.42
5	0.5	0	0.82	0.82	n/a	1.00

**Table 4:** Representative comparison between analytical and FEA-derived effective modulus values for GAN-CAD lattice candidates.

Scenario	Porosity P (%)	Normalized Kurtosis, K = Δ/a	E_Analytical (GPa)	E_FEA GAN-CAD (GPa)	Relative Error δ (%)
A1	89	0	4.12	3.96 ± 0.14	3.88
A2	89	0.3	1.90	1.98 ± 0.12	4.28
A3	65	0	16.48	15.80 ± 0.45	4.13
A4	65	0.5	2.64	2.77 ± 0.15	4.92
A5	35	0	37.08	35.64 ± 0.72	3.88
A6	35	0.5	13.35	14.22 ± 0.61	6.52

**Table 5:** Digital validation of elastic strain capacity and apparent Poisson response for selected GAN-CAD models.

P (%)	Δ/a	ε_an, Equation (8) (%)	ε_FEA (%)	ν_an	ν_FEA
89	0	0.82	0.81 ± 0.01	0.12	0.11 ± 0.01
89	0.5	20.5	20.36 ± 0.44	−0.10	−0.07 ± 0.01
65	0.5	5.12	5.02 ± 0.10	−0.025	−0.04 ± 0.01

## Data Availability

The analytical model, the trained conditional GAN and its training configuration, the generated voxel datasets, the reconstructed CAD models, and the finite-element input files that support the findings of this study are available from the corresponding author upon reasonable request.
